# SPINet: structure-enhanced point cloud instance segmentation network for multi-plant rice phenotyping

**DOI:** 10.3389/fpls.2026.1872119

**Published:** 2026-09-14

**Authors:** Wentao Song, Fang Qu, Yuwei Hao, He Huang, Caiguo Tang, Youqiang Sun, Junqing Zhang

**Affiliations:** 1 Hefei Institutes of Physical Science, Chinese Academy of Sciences, Hefei, China; 2 University of Science and Technology of China, Hefei, China; 3 Institute of Hefei Artificial Intelligence Breeding Accelerator Co. Ltd., Hefei, China

**Keywords:** 3D instance segmentation, grain counting, Mamba2, multi-plant scene, point cloud, rice phenotyping, spatial feature learning

## Abstract

Accurate 3D phenotyping of rice in multi-plant scenarios is important for evaluating yield-related traits and supporting high-throughput breeding. In particular, grain-level instance segmentation from point clouds provides a direct basis for quantifying panicle structure and grain number. However, this task remains challenging because densely grown rice plants often exhibit severe inter-plant occlusion, organ adhesion, and highly similar local structures, making it difficult to simultaneously capture global plant organization and fine-grained grain geometry. To address these challenges, we propose SPINet, a structure-enhanced point cloud instance segmentation network for 3D phenotypic extraction in multi-plant rice scenes. The proposed framework introduces a RiceMamba2 encoder that combines the long-range dependency modeling capability of Mamba2 with a parallel convolutional branch for sequence-neighborhood enhancement, enabling effective representation of both global spatial context and local organ-level details. In addition, a center prior-guided decoder is developed to provide explicit spatial anchors for instance queries, thereby improving the separation of adjacent and adhered rice organs in dense scenes. To further enhance training stability and convergence, a DINO-based denoising strategy is incorporated into the optimization process. Experimental results on the controlled indoor Grouped Rice Dataset (GRD) show that SPINet achieves an average precision (AP) of 65.73%, outperforming OneFormer3D by 31.38 percentage points. Furthermore, grain counting based on the predicted instances obtains a coefficient of determination (*R*^2^) of 0.8532, demonstrating the potential of SPINet for downstream yield-related phenotypic trait extraction. These results suggest that SPINet provides a promising framework for automated 3D rice phenotyping in complex multi-plant scenarios.

## 1 Introduction

Rice is one of the most important staple crops worldwide. Improving its yield potential remains a central objective of modern crop breeding and food security research. Yield formation is closely associated with structural and reproductive traits, including plant architecture, panicle morphology, grain number, and the spatial distribution of organs within the canopy. Accurate phenotypic characterization of these traits provides an essential basis for understanding genotype–environment interactions, evaluating germplasm resources, and selecting superior breeding materials (Gill et al., 2022). However, conventional phenotypic measurements often rely on manual observation or destructive sampling. These approaches are laborintensive, time-consuming, and difficult to scale to large breeding populations.

With the development of high-throughput phenotyping technologies, three-dimensional (3D) sensing has provided new opportunities for non-destructive crop trait measurement. Compared with two-dimensional (2D) images, which are often affected by viewpoint dependency, illumination variation, and incomplete spatial representation, 3D point clouds explicitly describe crop morphology and spatial organization. They are therefore well suited for quantifying complex plant traits such as plant height, leaf area, organ architecture, panicle structure, and canopy distribution (Paulus, 2019; Thapa et al., 2018). In rice phenotyping, 3D point clouds are particularly valuable because they preserve depth information and can better represent the spatial arrangement of panicles and grains under dense planting conditions.

Instance segmentation of plant point clouds is a critical step toward organ-level and grain-level phenotypic analysis. By separating individual organs or yield-related structures from the whole point cloud, instance segmentation enables subsequent measurement of biologically meaningful traits, such as grain number, grain size, panicle compactness, and organ spatial distribution. Early deep learning methods, such as PointNet (Qi et al., 2017a) and PointNet++ (Qi et al., 2017b), directly process unordered point sets and have promoted the development of point cloud-based plant analysis. However, their ability to capture complex local geometry remains limited. Sparse convolutional networks, represented by MinkowskiNet (Choy et al., 2019), improve computational efficiency by operating only on occupied voxels and have been applied to large-scale crop point clouds, including maize, sorghum, and cotton (Li et al., 2022a; Luo et al., 2023). In parallel, Transformer-based architectures have shown strong potential in plant point cloud segmentation because of their ability to model long-range dependencies. For example, PST was designed for plant point clouds and achieved promising results in rapeseed segmentation (Du et al., 2023), while Plant-MAE introduced masked autoencoding to reduce the dependence on large-scale manual annotations (Xie et al., 2025).

Despite these advances, most existing studies have focused on individual plants, isolated organs, or relatively sparse scenes. In practical rice breeding and phenotyping scenarios, plants are usually grown in groups or dense stands, where neighboring plants overlap, leaves and panicles intertwine, and grains are spatially clustered. When the analysis shifts from single-plant structures to multi-plant rice scenes, and the segmentation target extends to yield-critical grain-level structures, the task becomes substantially more challenging. In such dense scenes, point clouds from different plants or organs are often spatially close or even adhered, making it difficult to determine whether neighboring points belong to the same biological structure or to different instances. Traditional clustering-based methods are highly sensitive to this spatial adhesion; for example, the accuracy of individual maize plant segmentation was reported to decline markedly under dense planting conditions (Miao et al., 2021). Although PlantSegNet extended instance segmentation to outdoor field environments, its performance still depends strongly on semantic segmentation quality and remains vulnerable to ambiguous instance boundaries (Zarei et al., 2024). These limitations indicate that robust 3D instance segmentation in multi-plant rice scenes remains a key bottleneck for automated grain-level phenotyping.

Two challenges are particularly important from both agricultural and computational perspectives. First, rice point clouds contain hierarchical structural information, including the global arrangement of plants and panicles, as well as fine-grained local details of grains and organ boundaries. For yield-related phenotyping, preserving these local grain structures is essential because small segmentation errors can directly affect grain counting and trait estimation. Transformer-based encoders can capture global context, but their quadratic complexity often requires substantial downsampling, which may remove fine geometric details (Du et al., 2023; Xie et al., 2025). Sparse convolutional networks preserve local geometry more efficiently, but their limited receptive field restricts long-range dependency modeling (Choy et al., 2019; Li et al., 2022a). Therefore, an effective encoder for multi-plant rice phenotyping should jointly model global canopy-level structure and local grain-level geometry without excessive loss of point-level detail. Second, inter-plant occlusion and organ adhesion lead to ambiguous instance attribution. In occluded regions, grains or panicles from different plants may show similar geometry and continuous spatial distributions, resulting in instance merging, splitting, or boundary misclassification. These errors directly reduce the reliability of downstream traits such as grain number, panicle compactness, and yield-related measurements.

Recent advances in State Space Models (SSMs) provide a promising direction for efficient global modeling of large-scale point clouds. Mamba (Gu and Dao, 2024) and Mamba2 (Dao and Gu, 2024) achieve long-range sequence modeling with linear computational complexity, making them attractive for high-density 3D crop data. Several studies have begun to explore their potential in point cloud and 3D vision tasks, including PointMamba (Liang et al., 2024), VoxelMamba (Zhang et al., 2024), and Mamba3D (Han et al., 2024). However, pure SSM-based architectures are not inherently optimized for capturing local geometric details, which are essential for distinguishing adhered grains and delineating organ boundaries. Meanwhile, query-based segmentation frameworks inspired by DETR (Carion et al., 2020) provide an end-to-end paradigm for object and instance prediction. Subsequent methods, including Deformable DETR (Zhu et al., 2021), DAB-DETR (Liu et al., 2022), and RT-DETR (Zhao et al., 2024), improved convergence and efficiency, and this paradigm has been extended to 3D instance segmentation by methods such as Mask2Former (Cheng et al., 2022), Mask3D (Schult et al., 2023), SPFormer (Sun et al., 2023), and OneFormer3D (Kolodiazhnyi et al., 2024). Nevertheless, Hungarian matching in query-based models can be unstable in dense small-instance scenarios. Denoising training strategies, such as DN-DETR (Li et al., 2022b) and DINO (Zhang et al., 2023), alleviate this issue by introducing auxiliary noisy queries, but their integration with agricultural geometric priors, such as the spatial centers of crop organs, remains insufficiently explored.

To address these challenges, this study proposes SPINet, a structure-enhanced point cloud instance segmentation network for 3D phenotypic extraction in multi-plant rice scenes. SPINet is a coordinated integration of three key designs:

- A RiceMamba2 encoder is proposed for high-density rice point clouds. By integrating the long-range modeling capability of Mamba2 with a parallel convolutional branch, the encoder jointly captures global plant-level spatial context and short-range sequence-neighborhood features.
- A center prior-guided decoder is designed to improve instance discrimination under inter-plant occlusion and organ adhesion. By introducing geometric center priors into the query process, the decoder provides explicit spatial anchors for separating adjacent grains and panicles in dense rice scenes.
- A DINO-based denoising training strategy is incorporated to stabilize query learning and accelerate convergence. This improves the robustness of SPINet when handling numerous small, similar, and closely distributed grain instances in multi-plant rice point clouds.

## 2 Materials and methods

### 2.1 Data acquisition and dataset construction

The Grouped Rice Dataset (GRD) was constructed to support 3D instance segmentation and phenotypic analysis in multi-plant rice scenes. The dataset contains reconstructed RGB point clouds, multi-view images acquired from a 360^°^ surrounding trajectory, and the corresponding camera projection parameters. Instance-level annotations were provided for three organ categories, namely grain, leaf, and stem.

As shown in **Figure 1**, the data acquisition system consisted of a JAKA robotic arm equipped with an Intel RealSense D435i RGB-D camera. During acquisition, the robotic arm was controlled to move along a circular trajectory around the rice plants, enabling complete 360^°^ multi-view image capture. For each scene, a 20-second video was recorded at a resolution of 1920×1080 pixels and a frame rate of 30 fps. Keyframes were then extracted at 6 fps, resulting in approximately 120 multi-view images for each reconstructed sample.

**Figure 1:**
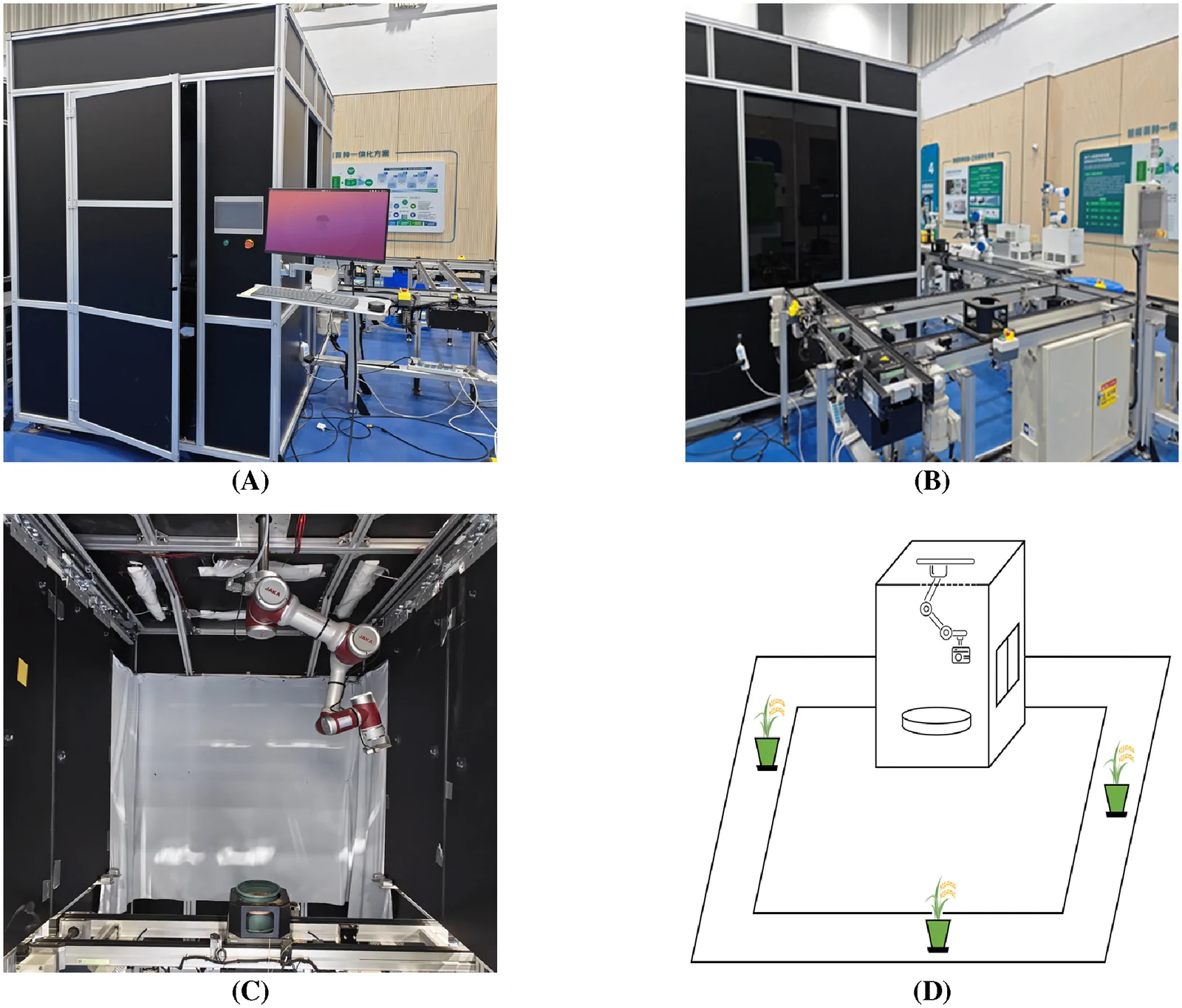
Phenotyping box and data acquisition pipeline used for constructing the Grouped Rice Dataset (GRD). **(A)** Front view of the phenotyping box. **(B)** Side view of the phenotyping box. **(C)** Interior view showing the imaging setup. **(D)** Workflow of multi-view image acquisition, 3D reconstruction, point cloud export, and instance annotation.

The overall NeRF-based 3D reconstruction framework is illustrated in **Figure 2**. After image acquisition, COLMAP (Schönberger and Frahm, 2016) was used to estimate the camera poses and extrinsic parameters from the extracted multi-view images. Based on the recovered camera parameters, Nerfacto (Tancik et al., 2023) was employed to learn a continuous volumetric representation of each rice scene. The reconstructed radiance field was subsequently converted into RGB point clouds, which were used as the input data for instance segmentation and downstream phenotypic analysis.

**Figure 2:**
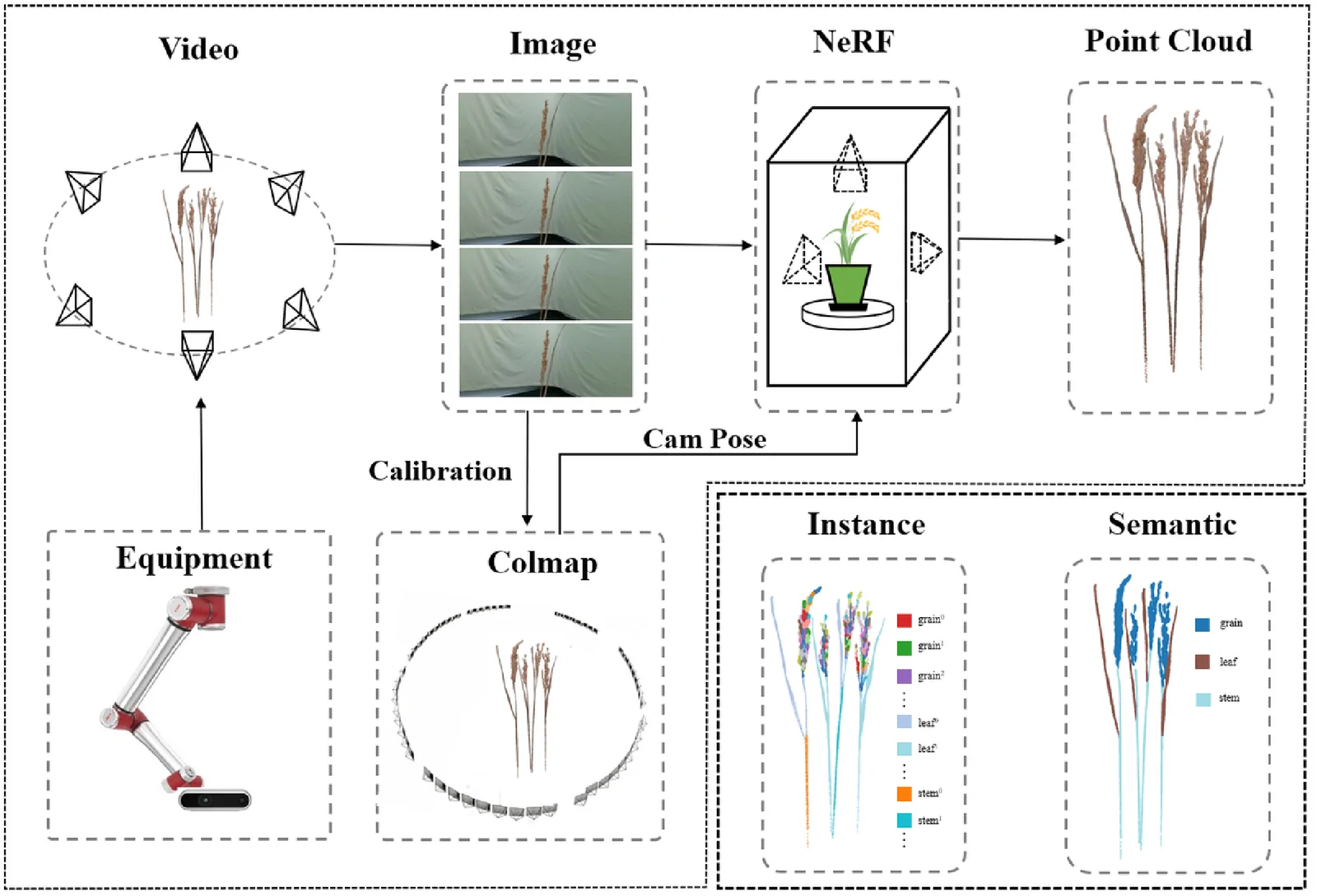
NeRF-based 3D reconstruction framework for rice phenotyping. Multi-view images were first used for camera pose estimation with COLMAP, followed by Nerfacto reconstruction and RGB point cloud export for instance segmentation and phenotypic analysis.

For point cloud preprocessing, each reconstructed sample initially contained approximately 100,000 points with XYZRGB information, including 3D coordinates and color attributes. To improve data quality, statistical outlier removal based on a Gaussian distribution assumption was applied to eliminate isolated noisy points. The point clouds were then downsampled to a consistent density while preserving grain-level geometric details, ensuring that the final grouped rice point clouds retained sufficient spatial resolution for fine-grained instance segmentation.

Instance-level annotation was conducted in CloudCompare by assigning each point a semantic category and instance ID for grain, leaf, or stem. The resulting annotations were saved in TXT format and paired with the reconstructed RGB point clouds to construct the GRD dataset. Detailed statistics are provided in **Table 1**.

**Table 1:** Statistics of the GRD dataset.

Dataset	Number of plants	Organ category	Number of instances
GRD	150	Grain	5382
		Leaf	158
		Stem	150

### 2.2 SPINet: network architecture

This section details the SPINet (Structure-enhanced Point Cloud Instance Segmentation Network) framework, designed to address the challenges of acquiring high-precision plant phenotypic information in multi-plant scenarios. As illustrated in **Figure 3**, SPINet is an end-to-end instance segmentation network that receives high-density point clouds reconstructed by NeRF as input. The network is sequentially optimized through three main components: the RiceMamba2 encoder, a query-based decoder with geometric center priors, and a joint loss function incorporating the DINO denoising concept. This process ultimately outputs high-precision instance segmentation results. The design of the entire framework aims to balance the efficiency and accuracy of large-scale point cloud processing, while addressing the segmentation inaccuracy caused by occlusion among multiple rice plants. Its core comprises the following three innovative modules:

**Figure 3:**
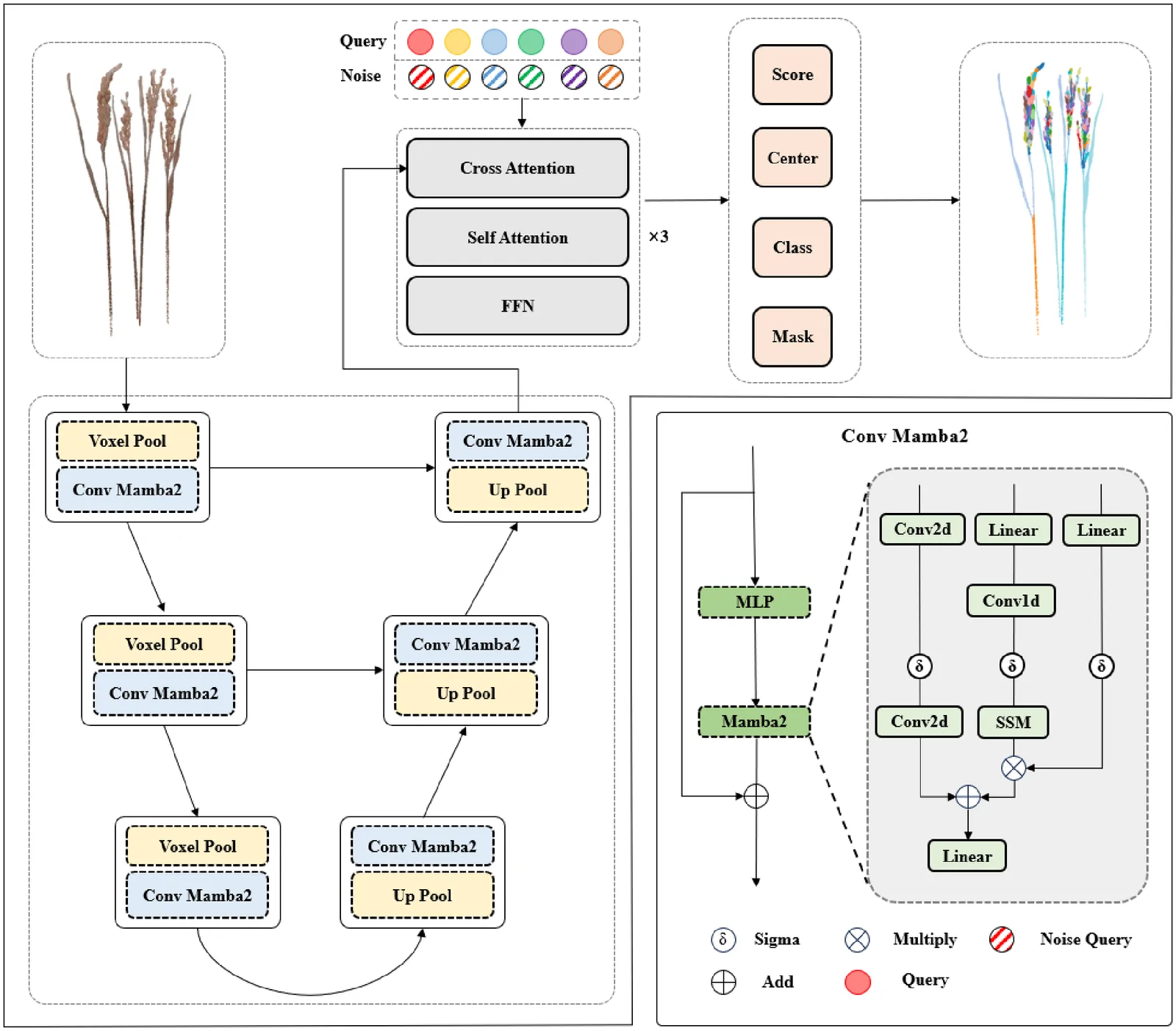
Overall architecture of SPINet for 3D point cloud instance segmentation in multi-plant rice scenarios.

1. RiceMamba2 Encoder: To address the difficulty of existing feature extraction networks in simultaneously capturing global and local spatial information, the RiceMamba2 backbone network was designed based on the Mamba2 architecture. This network introduces a parallel convolutional branch alongside the Mamba2 backbone structure, and integrates the outputs of both through a feature fusion mechanism, enabling the encoder to model both global context and short-range correlations among neighboring feature indices while maintaining linear computational complexity, thereby meeting the efficient processing requirements of large-scale point clouds (e.g., exceeding 100,000 points per plant).
2. Center-guided Instance Decoder: To address the ambiguous instance attribution caused by inter-plant occlusion, a Transformer decoder based on geometric center priors is proposed. This decoder no longer employs randomly initialized or purely content-driven queries; instead, it introduces the geometric centers of instances as spatial anchors into the query initialization process, thereby simplifying the complex instance search task into boundary regression based on given centers, significantly improving the instance discrimination capability in dense scenarios.
3. Denoising-aided Matching Criterion: To accelerate model convergence and improve robustness, a novel loss function was designed by incorporating the denoising concept from DINO. This function actively adds noise to the center points and category labels of instances during training, forcing the model to learn more discriminative instance-level features, thereby reaching a stable state more rapidly during the optimization process.

#### 2.2.1 Problem formulation

The objective of 3D instance segmentation is to partition a point cloud scene into a set of non-overlapping instances and assign a semantic category to each instance. Given an input point cloud $P={\left\{p_{i}\right\}}_{i=1}^{N}\subset {\mathbb{R}}^{3+d}$, where *N* denotes the number of points and each point *p_i_*consists of its 3D coordinates and a *d*-dimensional attribute vector, the task is to learn the mapping function defined in Equation (1):

(1) f:P→Y.

The output of the mapping function is defined in Equation (2):

(2) $$Y={\left\{(M_{k},c_{k})\right\}}_{k=1}^{K},$$

where *K* is the number of predicted instances, $M_{k}\subset P$ denotes the point mask of the *k*-th instance, and $c_{k}\in C$ represents its semantic category. The category set *C* includes grain, leaf, and stem in this study. The predicted instance masks are expected to satisfy the mutual-exclusion constraint in Equation (3):

(3) $$M_{i}\cap M_{j}=\emptyset ,\;\forall i\neq j.$$

Therefore, the goal of SPINet is to predict both the instance identity and semantic category for each point, enabling fine-grained organ-level segmentation and subsequent phenotypic trait extraction.

#### 2.2.2 Preliminaries

High-resolution point clouds are required for grain-level phenotypic analysis because dense and small rice organs contain fine geometric details that are easily lost during aggressive downsampling. However, existing point cloud feature extraction networks face a fundamental trade-off between local geometric representation and global spatial modeling.

Sparse convolutional networks, such as MinkowskiNet (Choy et al., 2019), process only occupied voxels and therefore provide an efficient solution for large-scale point clouds. They are effective in preserving local spatial structures, but their receptive fields are inherently constrained by the convolutional hierarchy, making it difficult to directly model long-range dependencies across different plant organs. In contrast, self-attention-based architectures can capture global context self-attention-based architectures can capture global context through the pairwise interactions formulated in Equation (4):

(4) $$\text{Attention}(Q,K,V)=\text{softmax}(\frac{QK^{T}}{\sqrt{d_{k}}})V.$$

Nevertheless, the quadratic complexity of self-attention with respect to the number of points limits its applicability to high-density rice point clouds, where excessive downsampling may remove fine-grained grain structures.

State Space Models (SSMs) offer an alternative route for efficient long-range modeling. A discrete SSM maps an input sequence *X_t_*to an output sequence *Y_t_*through a hidden state *h_t_*, as formulated in Equation (5):

(5) $$h_{t}=\bar{A}h_{t-1}+\bar{B}X_{t},\;Y_{t}=Ch_{t}+DX_{t}.$$

Mamba further introduces an input-dependent selective mechanism, allowing the model parameters to adapt dynamically to the input sequence. Mamba2 improves the computational efficiency of this framework through State Space Duality (SSD), which reformulates sequence modeling into an attention-like dual form shown in Equation (6):

(6) $$(L\circ QK^{⊤})V,\;L_{ij}=\{\begin{array}{ll} a_{i}\times \cdots \times a_{j+1}, & i\geq j,\\ 0, & i<j. \end{array}$$

This formulation enables efficient matrix multiplication while retaining linear-complexity sequence modeling.

Although recent Mamba-based 3D methods, including PointMamba (Liang et al., 2024), VoxelMamba (Zhang et al., 2024), and Mamba3D (Han et al., 2024), have demonstrated the potential of SSMs for point cloud representation learning, they mainly emphasize global sequence modeling and provide limited explicit modeling of sequence-neighborhood enhancement. This limitation is particularly critical for rice point clouds, where fine-grained grain morphology and adhered organ boundaries are essential for accurate instance segmentation. Motivated by this observation, we develop the RiceMamba2 encoder, which augments Mamba2 with a parallel convolutional branch to enhance short-range sequence-index correlations while preserving efficient long-range dependency modeling. The detailed design of RiceMamba2 is described below.

#### 2.2.3 RiceMamba2 encoder

Mamba2 provides an efficient mechanism for long-range dependency modeling with linear computational complexity. However, existing Mamba-based point cloud methods mainly emphasize global sequence modeling and lack explicit mechanisms for extracting short-range sequence-index correlations. This limitation is critical for 3D instance segmentation of rice point clouds, where fine-scale features such as edges, curvature, and local surface variations are essential for distinguishing adhered organs and accurately delineating instance boundaries. To address this issue, we propose the RiceMamba2 encoder, which augments the Mamba2 backbone with a parallel convolutional branch to jointly model global context and local geometric details.

The overall design of RiceMamba2 is illustrated in the bottom-right part of **Figure 3**. Given an input feature sequence, the module adopts a three-branch structure. The first two branches follow a gated Mamba2-based formulation to capture global dependencies, whereas the third branch explicitly enhances short-range sequence-index correlations through convolutional operations.

The first branch takes the first half of the input feature, denoted as $F_{b_{1}}\in {\mathbb{R}}^{L\times \frac{C}{2}}$, and generates a gating signal through a linear projection followed by a SiLU activation, as defined in Equation (7):

(7) $${\tilde{F}}_{1}=\text{SiLU}(\text{Linear}(F_{b_{1}})).$$

The second branch processes the remaining half of the input feature, denoted as $F_{b_{2}}\in {\mathbb{R}}^{L\times \frac{C}{2}}$. It first applies a linear projection and one-dimensional convolution, and then uses the SSM module of Mamba2 to model long-range dependencies. Layer normalization is subsequently applied to stabilize feature representation, as defined in Equation (8):

(8) $${\tilde{F}}_{2}=\text{LN}(\text{SSM}(\text{Conv}1\text{D}(\text{Linear}(F_{b_{2}})))).$$

The outputs of these two branches are fused using the Hadamard product, allowing the gating branch to modulate the global contextual features extracted by the Mamba2 branch. This design enhances the selectivity of global feature propagation and improves the representation of long-range spatial relationships.

The third branch is introduced to complement the long-range modeling capability of the Mamba-based architecture by enhancing short-range interactions among neighboring feature indices. The input feature is rearranged into a spatial feature representation $F_{b_{3}}\in {\mathbb{R}}^{C\times H\times W}$, where short-range sequence-index correlations can be captured through convolutional operations. Two convolutional layers with a SiLU activation are then used to extract local geometric features, as formulated in Equation (9):

(9) $${\tilde{F}}_{3}=\text{Conv}2\text{D}(\text{SiLU}(\text{Conv}2\text{D}(F_{b_{3}}))).$$

Finally, the gated global feature and the local convolutional feature are integrated through element-wise addition, followed by a linear projection to produce the global-local feature representation, as defined in Equation (10):

(10) $$F_{gl}=\text{Linear}({\tilde{F}}_{1}⊙{\tilde{F}}_{2}+{\tilde{F}}_{3}),$$

where ⊙ denotes the Hadamard product.

To further improve feature propagation and training stability, a scaled residual connection is used to combine the output of RiceMamba2 with the original input feature. Through this design, RiceMamba2 preserves the efficient long-range modeling ability of Mamba2 while explicitly strengthening short-range sequence-index correlations. The resulting features provide rich global semantics and fine-grained structural cues for the subsequent instance decoder, which is particularly important for separating dense and adhered rice organs in multi-plant scenes.

#### 2.2.4 Center prior-guided instance decoder

Severe inter-plant occlusion and high morphological similarity among rice organs often lead to ambiguous instance attribution and inaccurate boundary delineation in multi-plant scenes. To alleviate this problem, we introduce a center prior-guided instance decoder that explicitly incorporates the geometric centers of instances into the query formulation. The underlying motivation is that, although instance boundaries may become unclear in occluded or adhered regions, the geometric center of each independent instance usually remains spatially distinguishable and provides a stable cue for instance localization.

Inspired by DAB-DETR (Liu et al., 2022), each decoder query is formulated as a combination of semantic content and spatial position, enabling the model to answer both “what to segment” and “where to segment”. Specifically, each query consists of a learnable content query *Q_c_*and a positional query *Q_p_*. The content query encodes category-related information, whereas the positional query is generated from the 3D center coordinate of an instance through a positional encoding function. The final query representation is defined in Equation (11):

(11) $$Q=Q_{c}+Q_{p}.$$

By providing explicit spatial anchors, the decoder narrows the instance search space from the entire scene to local regions around potential instance centers. This center-aware formulation helps reduce instance merging and splitting errors caused by inter-plant occlusion and improves the discrimination of adjacent or adhered rice organs.

In this study, the geometric center of an instance is defined as the center of its 3D oriented bounding box (OBB). Given an instance point set $P={\left\{p_{i}\right\}}_{i=1}^{N}$, where $p_{i}=(x_{i},y_{i},z_{i})$ denotes the coordinate of the *i*-th point, the centroid is first computed according to Equation (12):

(12) $$C=\frac{1}{N}\sum_{i=1}^{N}p_{i}$$

Principal component analysis (PCA) is then applied to the covariance matrix of the instance point set to obtain three orthogonal principal axes **v**_1_, **v**_2_, and **v**_3_. All points are projected onto each principal axis, and the projection boundaries are computed according to Equation (13):

(13) $$\underset{k}{\min}=\underset{i}{\min}(p_{i}\cdot v_{k}),\;\underset{k}{\max}=\underset{i}{\max}(p_{i}\cdot v_{k}),\;k\in \{1,2,3\}.$$

The size of the OBB and its center in the local PCA coordinate system are then obtained using Equation (14):

(14) $$d_{k}=\underset{k}{\max}-\underset{k}{\min},\;C_{\text{box}}^{\left(k\right)}=\frac{\max_{k}+\min_{k}\;}{2},\;k\in \left\{1,2,3\right\}.$$

The resulting OBB center is used as the geometric center prior for query initialization during training.

**Figure 4** illustrates the OBB-based center estimation for rice grains.

**Figure 4:**
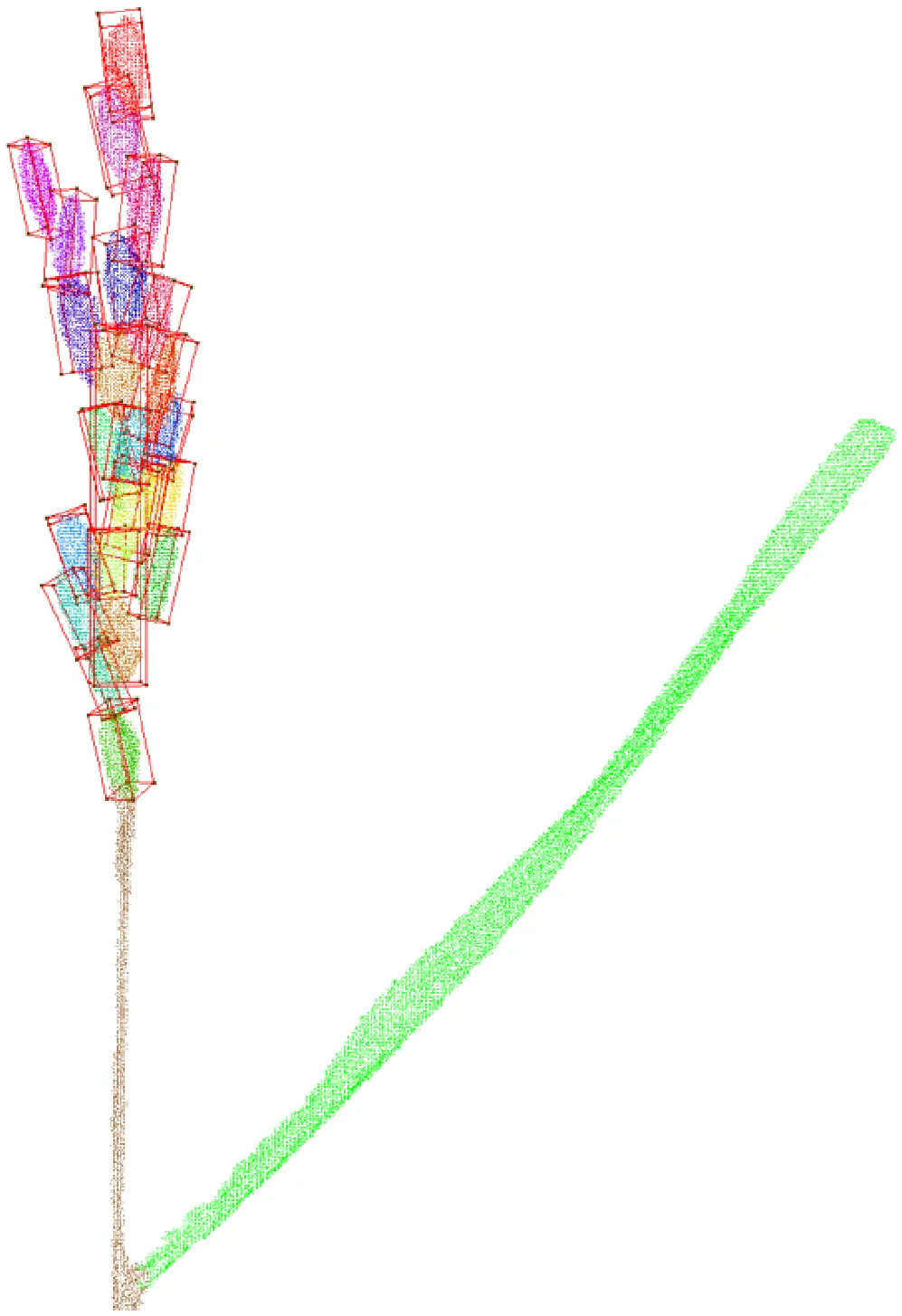
OBB-based geometric center estimation for rice grain instances. The oriented bounding box provides a stable spatial prior for initializing center-guided decoder queries.

To avoid over-reliance on ground-truth centers, the regular instance queries use learnable reference points during both training and inference. Specifically, the decoder maintains *K* = 400 learnable reference points, $R^{\left(0\right)}\in {\left[0,1\right]}^{K\times 3}$, where each row represents a normalized three-dimensional coordinate. The three coordinate components are independently initialized from a uniform distribution *U*(0,1) and are optimized jointly with the remaining network parameters. In parallel, the semantic content of the regular queries is represented by a learnable embedding $Q_{c}\in {\mathbb{R}}^{K\times D}$, where *D* = 256.

The normalized reference coordinates are transformed into positional embeddings using a two-layer multilayer perceptron defined in Equation (15):

(15) $$Q_{p}^{\left(0\right)}=\phi_{\text{pos}}(R^{\left(0\right)}),\;Q^{\left(0\right)}=Q_{c}+Q_{p}^{\left(0\right)},$$

where *ϕ*_pos_(·) denotes the positional encoding network and **Q**^(0)^ represents the initial instance queries. In this way, each query combines a learnable semantic component with an explicit normalized spatial anchor before entering the decoder.

The decoder contains *L* = 3 stacked refinement layers. At the *l*-th layer, the query features are successively updated through cross-attention with the encoder features, self-attention among the instance queries, and a feed-forward network. A shared center-offset head then predicts a three-dimensional residual Δ**R**^(^*^l^*^)^ from the updated query features. Instead of directly regressing an unconstrained coordinate, the reference point is refined in the inverse-sigmoid space according to Equation (16):

(16) $$R^{\left(l\right)}=\text{sigmoid }(\text{Δ}R^{\left(l\right)}+\text{logit }(R^{\left(l-1\right)})),\;l=1,\ldots ,L,$$

where logit(·) is the inverse of the sigmoid function. This residual update preserves the normalized coordinate range while allowing each decoder layer to progressively correct the preceding center estimate.

For center prediction, the normalized reference coordinates are mapped back to the coordinate range of the current point cloud. Let **p**_min_ and **p**_max_ denote the minimum and maximum XYZ coordinates of the input scene, respectively. The center prediction at decoder layer *l* is obtained using Equation (17):

(17) $$C_{\text{pred}}^{\left(l\right)}=R^{\left(l\right)}⊙(p_{\max\;\;}-p_{\min\;\;})+p_{\min\;\;},$$

where ⊙ denotes element-wise multiplication. The refined reference coordinates are detached before being passed to the next refinement layer, as shown in Equation (18):

(18) $${\tilde{R}}^{\left(l\right)}=\text{stopgrad }(R^{\left(l\right)}).$$

The detached coordinates serve as residual anchors for the center-offset prediction of the subsequent layer. This operation prevents gradients from later refinement stages from being propagated recursively through all preceding coordinate updates and improves optimization stability.

During layer-wise decoding, the query features are updated through the attention and feed-forward operations, whereas the reference coordinates follow a parallel iterative refinement pathway through the center-offset head. The final-layer query features are used to predict instance categories, masks, and confidence scores, while $C_{\text{pred}}^{\left(L\right)}$ is used as the final center prediction.

During training, the *K* regular learnable queries follow the same initialization and refinement pathway as that used during inference. Additional denoising queries constructed from ground-truth instance centers are concatenated with the regular queries only as auxiliary training supervision, as described in Section 2.2.5. These denoising queries are removed during inference and therefore do not replace the learnable reference points of the regular queries.

The complete reference-point initialization and refinement procedure is summarized in **Algorithm 1**.

Algorithm 1. Reference-point refinement in the center-guided decoder.

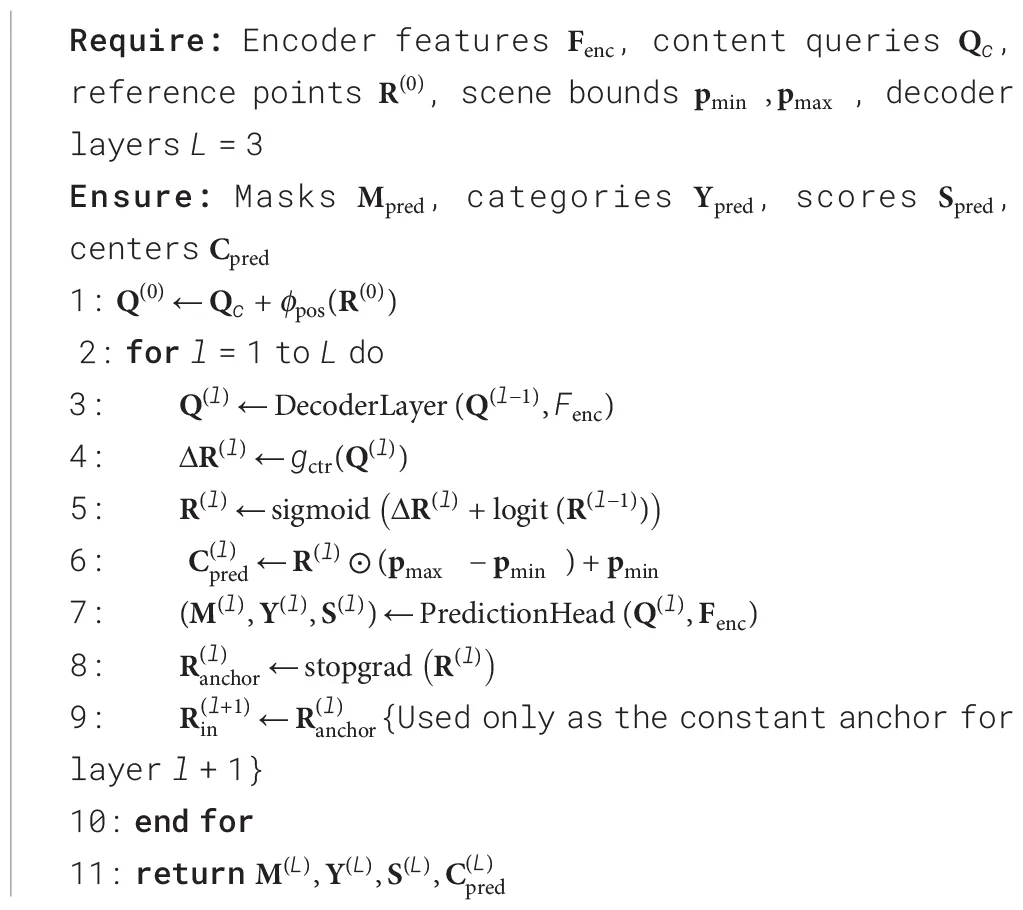

The stop-gradient operation is applied only to the reference-point state passed to the next refinement layer. The center prediction at layer *l* is computed before this operation and remains differentiable with respect to the layer-*l* center-offset head and query features. Therefore, the center regression loss at the current layer continues to supervise its center prediction. The stop-gradient operation only prevents losses from subsequent refinement layers from back-propagating recursively through the reference-point update at layer *l*. The classification, mask, and confidence-score predictions remain differentiable through the query features.

#### 2.2.5 DINO-based contrastive denoising and loss optimization

Although the geometric center-guided decoder provides explicit spatial priors for instance queries, the standard Hungarian matching strategy used in DETR-like models (Carion et al., 2020; Zhu et al., 2021) may still suffer from unstable optimization during the early training stage. This problem becomes more pronounced in dense multi-plant rice point clouds, where numerous small grain instances are spatially close and visually similar. As illustrated in **Figure 5**, unstable matching may cause multiple queries to compete for the same target, leading to duplicate predictions, while small grain instances may receive insufficient or inconsistent supervision.

**Figure 5:**
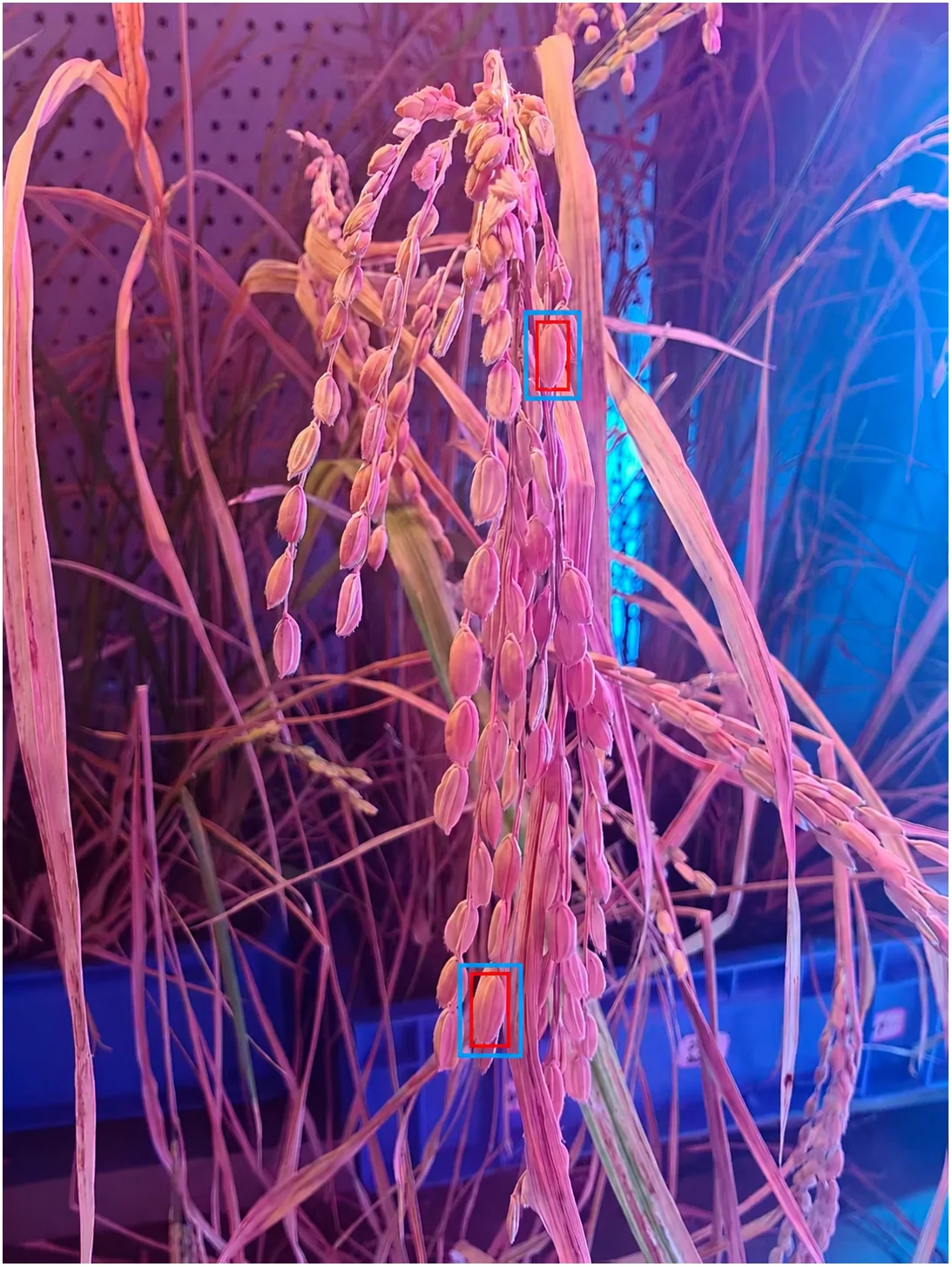
Duplicate predictions caused by unstable Hungarian matching in dense small-instance scenarios.

To improve matching stability and accelerate convergence, we incorporate the contrastive denoising training strategy inspired by DINO. As shown in **Figure 6**, the key idea is to construct auxiliary denoising queries from ground-truth instances. These queries are generated by perturbing the ground-truth center coordinates and category labels, allowing the model to learn not only how to recover valid targets from slightly corrupted queries, but also how to suppress heavily corrupted or mismatched queries. In this way, the denoising task provides direct and stable supervision before the regular matching queries become sufficiently reliable.

**Figure 6:**
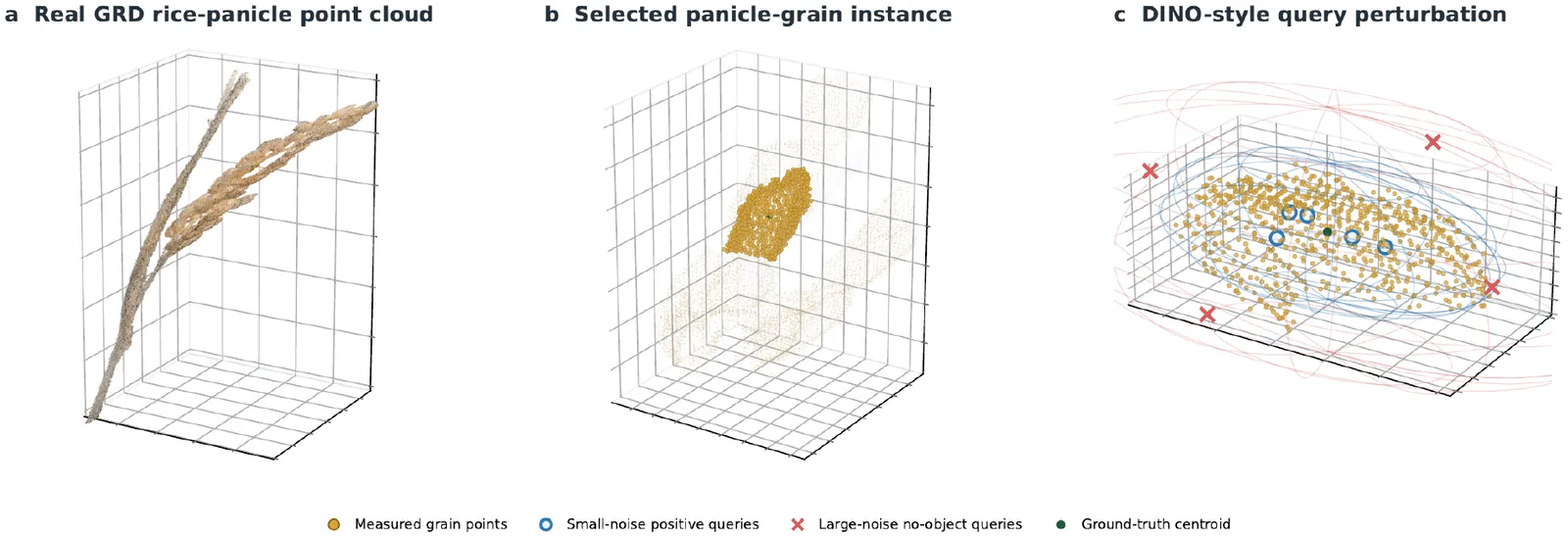
DINO-based contrastive denoising strategy. **(a)** Real GRD rice-panicle point cloud containing panicle branches and grains. **(b)** A selected panicle-grain instance used to construct denoising queries. **(c)** DINO-style query perturbation, where positive denoising queries are generated by applying small perturbations around the ground-truth instance centroid and are supervised to reconstruct the corresponding ground-truth instance, whereas negative queries with larger perturbations are encouraged to predict the no-object class.

In the proposed framework, noise is added to both the geometric center and the category label of each ground-truth instance. For center perturbation, a random factor *ρ* ∼ *U*(−1,1) is sampled independently along the three spatial dimensions. Given the oriented bounding box dimensions (*w,l,h*) of an instance, the perturbed center offset is computed as:

(19) $$\begin{array}{ll} \text{Δ}x & =\rho_{x}\cdot \frac{w}{2}\cdot s_{\text{bbox}},\\ \text{Δ}y & =\rho_{y}\cdot \frac{l}{2}\cdot s_{\text{bbox}},\\ \text{Δ}z & =\rho_{z}\cdot \frac{h}{2}\cdot s_{\text{bbox}}, \end{array}$$

where *s*_bbox_ denotes the noise scale factor. Following DINO, a smaller noise scale is used to generate positive denoising queries, while a larger noise scale is used to generate negative queries. In our implementation, the positive and negative noise scales are set to $s_{\text{bbox}}^{pos}=0.2$ and $s_{\text{bbox}}^{neg}=1.0$, respectively.

For label perturbation, the ground-truth category label is randomly replaced by another category with a predefined probability. Specifically, for each instance, a random value *p_i_*∼ *U*(0,1) is sampled. If *p_i_*is smaller than the label noise ratio, the corresponding category label is replaced by a randomly selected incorrect class. The noise-perturbed centers and labels are jointly encoded as denoising queries, which are concatenated with the regular matching queries and fed into the decoder.

To prevent information leakage between denoising queries and regular matching queries, an attention mask is introduced in the self-attention layers. The mask blocks regular matching queries from attending to denoising queries, ensuring that the denoising task serves only as auxiliary supervision during training. This design preserves the independence of the matching process while allowing the model to benefit from more stable denoising signals.

The overall training objective consists of a regular matching loss and a denoising loss. The total loss is formulated in Equation (20):

(20) $$L=\lambda_{cls}L_{cls}+\lambda_{mask}L_{mask}+\lambda_{score}L_{score}+\lambda_{focal}L_{focal}+\lambda_{box}L_{box},$$

where *L_cls_*, *L_mask_*, and *L_score_*denote the classification loss, mask loss, and confidence loss for regular matching queries, respectively. *L_focal_*denotes the focal classification loss for denoising queries, and *L_box_*represents the center regression loss between the predicted and ground-truth center coordinates. The weighting coefficients *λ_cls_*, *λ_mask_*, *λ_score_*, *λ_focal_*, and *λ_box_*balance the contributions of different loss terms.

The center regression loss *L*_box_ is implemented using the Smooth-L1 loss between the predicted center ${\hat{c}}_{i}$ and the ground-truth center **c***_i_*. The center regression loss is defined in Equation (21):

(21) $$L_{\text{box}}=\frac{1}{3N_{m}}\sum_{i=1}^{N_{m}}\sum_{d\in \left\{x,y,z\right\}}{\text{smooth}}_{L1}\left({\hat{c}}_{i,d}-c_{i,d}\right),$$

where *N_m_*is the number of matched positive queries. With the default parameter *β* = 1, the Smooth-L1 function is defined in Equation (22):

(22) $$smooth_{L1}(e)=\{\begin{array}{ll} 0.5e^{2}, & \left|e\right|<1,\\ \left|e\right|-0.5, & \text{otherwise}. \end{array}$$

The three center-coordinate dimensions are equally weighted.

The joint denoising and matching training procedure is summarized in **Algorithm 2**.

Algorithm 2. Joint denoising and matching training.

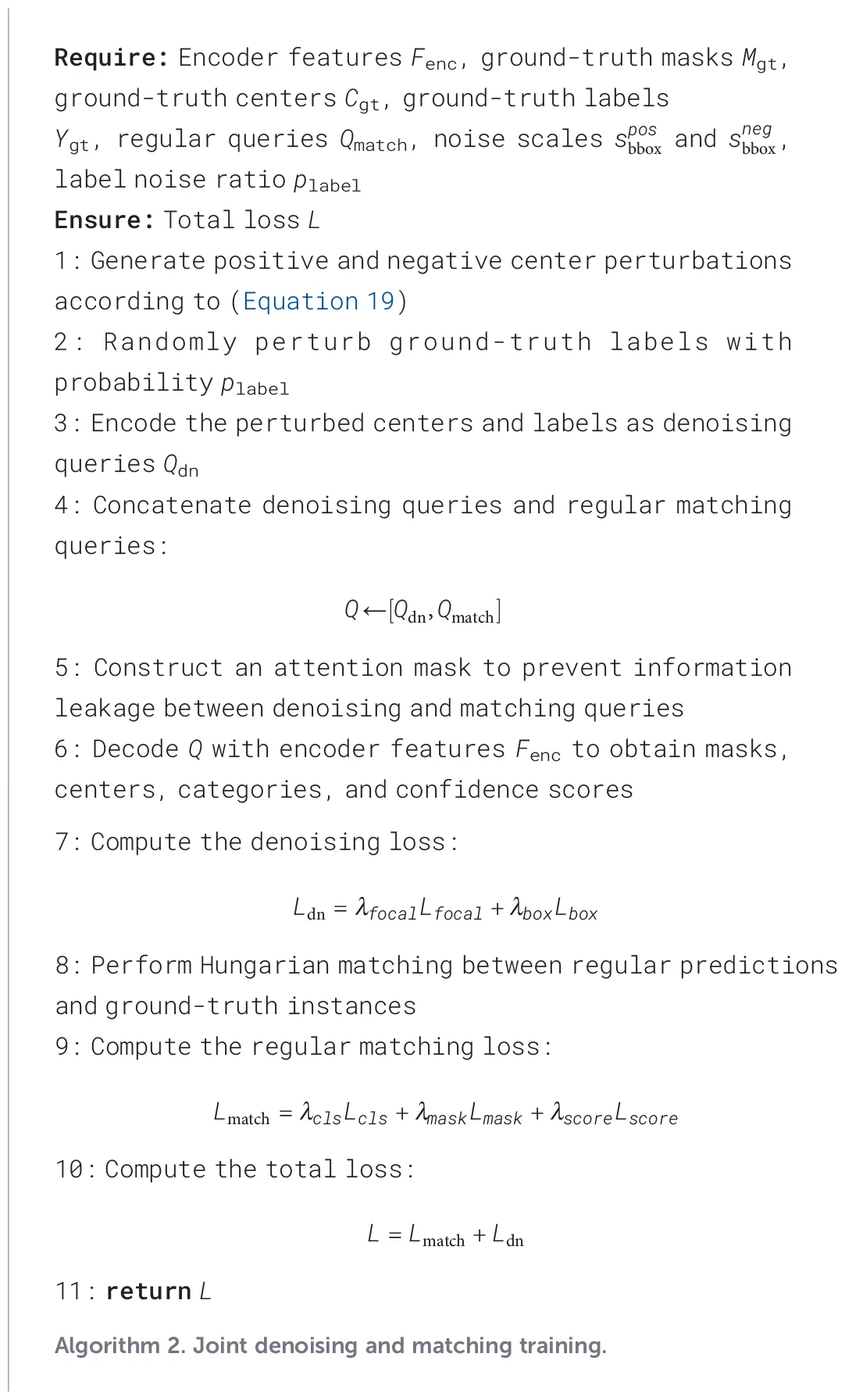

## 3 Results

### 3.1 Experimental setup

Dataset. Experiments were conducted on the GRD, which contains 150 annotated multi-plant rice point cloud scenes and 5,690 instance-level annotations. The dataset was divided into training and validation sets at a ratio of 8:2. Model performance was evaluated on three organ categories, namely leaf, stem, and grain. All quantitative results reported in this study are limited to the controlled indoor GRD setting. The current dataset does not provide independent field, cross-cultivar, or longitudinal validation.

Evaluation metrics. Following the COCO-style instance segmentation protocol (Lin et al., 2014), Average Precision (*AP*), *AP*_50_, and *AP*_25_ were used as the primary evaluation metrics. *AP* was computed by averaging the precision over IoU thresholds from 0.50 to 0.95 with an interval of 0.05, as formulated in Equation (23):

(23) $$AP=\frac{1}{10}\sum_{i=0}^{9}AP_{\text{IoU}=0.50+0.05i},$$

where *AP*_IoU=_*_t_*denotes the average precision at the IoU threshold *t*. *AP*_50_ and *AP*_25_ were additionally reported to evaluate segmentation performance under moderate and relaxed overlap criteria, respectively.

We further report the class-averaged instance recall at an IoU threshold of 0.50, denoted as *AR*_50_ (Recall@0.50), as defined in Equation (24):

(24) $$AR_{50}=\frac{1}{C}\sum_{c=1}^{C}\frac{TP_{c,50}}{TP_{c,50}+FN_{c,50}},$$

where *C* is the number of semantic categories, and *TP_c,_*_50_ and *FN_c,_*_50_ denote the numbers of matched and missed ground-truth instances of category *c* at an IoU threshold of 0.50, respectively. This metric follows the instance-segmentation evaluator used by OneFormer3D and should not be confused with the COCO detection AR averaged over IoU thresholds from 0.50 to 0.95.

Training configuration and implementation details. All experiments were conducted on a workstation equipped with one NVIDIA GeForce RTX 3090 GPU. The software environment included Python 3.8.19, PyTorch 1.13.1 with CUDA 11.6, cuDNN 8.5, TorchVision 0.14.1, OpenCV 4.9.0, MMEngine 0.10.3, MMDetection 3.3.0, and MMDetection3D 1.1.0 (MMDetection3D Contributors, 2020).

The model was trained using AdamW (Loshchilov and Hutter, 2019) with an initial learning rate of 0.0002 and a weight decay of 0.02. The batch size was set to 1, and gradient clipping with a maximum norm of 10 was applied. A polynomial learning-rate schedule with a power of 0.9 was adopted. The model was trained for 60,000 iterations, and validation was performed every 500 iterations. The complete training process for the selected run required approximately 6 h 38 min. The effective parameter count of SPINet was 34.357 M. The end-to-end validation time was approximately 0.1297 s per scene, corresponding to a throughput of 7.71 scenes/s.

Implemented loss configuration. The ten-term weight vector is *λ* = [0.5,1,1,1,1,0.5,0.5,0.5,0.5,0.5] for regular classification, regular/DN mask BCE, regular/DN Dice, regular/DN score MSE, DN focal classification, and regular/DN center regression. The center training losses use Smooth-*L*_1_ with *β* = 1 and mean reduction in scene coordinates, while Hungarian matching uses a separate *L*_1_ center cost computed using cdist(*p* = 1). The DN focal loss uses *γ* = 2 and *α* = 0.25.

Data augmentation. To improve model generalization, several point cloud augmentation strategies were applied during training, including horizontal flipping, random rotation around the vertical axis, elastic distortion, and random scaling. Since the proposed method directly performs instance segmentation on point clouds and superpoint preprocessing provided limited improvement in preliminary experiments, superpoint-related inputs were removed to simplify the training pipeline.

### 3.2 Quantitative analysis of 3D instance segmentation performance

#### 3.2.1 Effect of backbone networks

To evaluate the effectiveness of Mamba2 as the backbone network, we compared it with representative point cloud backbones, including SPUNet (Graham et al., 2018), Point Transformer v1 (Zhao et al., 2021), and Point Transformer v2 (Wu et al., 2022). As shown in **Table 2**, Mamba2 achieved the best performance across all evaluation metrics. Compared with the second-best backbone, SPUNet, Mamba2 improved *AP*, *AP*_50_, and *AP*_25_ by 25.01, 16.94, and 16.23 percentage points.

**Table 2:** Comparison of different backbone networks within the SPINet.

Framework	Backbone	*AP*	*AP*_50_	*AP*_25_
SPINet	SPUNet	32.68	51.50	64.18
	PTv1	25.79	37.11	59.27
	PTv2	26.65	49.04	69.23
	Mamba2	**57.69**	**68.44**	**80.41**

Bold values indicate the best results.

The superior performance of Mamba2 can be attributed to its ability to model long-range dependencies with linear computational complexity. This property is particularly beneficial for the GRD dataset, where each scene contains high-density point clouds and complex spatial relationships among multiple rice plants. In contrast, Point Transformer v2 relies on local attention windows, which may limit its ability to capture global topological relationships in large-scale point clouds. Point Transformer v1 showed the lowest *AP*, likely because its standard vector attention mechanism introduces high computational overhead and unstable optimization when processing dense point clouds. SPUNet achieved the second-best performance, indicating the effectiveness of sparse convolution for local structure extraction; however, its limited receptive field restricts long-range context modeling in occluded multi-plant scenarios.

#### 3.2.2 Comparison with 3D instance segmentation methods

The overall comparison with state-of-the-art 3D instance segmentation methods is presented in **Table 3**. SPINet achieved the highest *AP* and *AP*_50_ among all compared methods, reaching 65.73 and 75.04, respectively. Compared with OneFormer3D, SPINet improved *AP*, *AP*_50_, and *AP*_25_ by 31.38, 15.70, and 4.63 percentage points.

**Table 3:** Comparison of SPINet with state-of-the-art 3D instance segmentation methods on the GRD dataset.

Method	*AP*	*AP*_50_	*AP*_25_
PlantSegNet	1.63	3.78	5.30
MinkUNet-v2	10.02	25.48	38.28
SoftGroup	21.95	32.97	46.55
SoftGroup++	29.17	44.23	52.89
ISBNet	29.03	46.83	76.44
SPFormer	34.08	58.44	**83.94**
OneFormer3D	34.35	59.34	77.73
VoxelMamba	35.93	59.12	82.62
SPINet	**65.73**	**75.04**	82.36

Bold values indicate the best results.

It is worth noting that SPFormer obtained the highest *AP*_25_ value, slightly exceeding SPINet by 1.58 percentage points. Since *AP*_25_ uses a relaxed IoU threshold, this metric mainly reflects coarse instance localization rather than precise boundary delineation. In contrast, SPINet showed clear advantages under stricter evaluation criteria, particularly in *AP* and *AP*_50_. This indicates that SPINet not only detects rice organ instances effectively but also provides more accurate mask predictions and instance boundaries.

To compare whether a Mamba-style voxel encoder can directly improve an existing instance segmentation pipeline, we adapted VoxelMamba to OneFormer3D by replacing only the encoder while keeping all other settings fixed. The result achieved validation *AP* 35.93%, *AP*_50_ 59.12%, and *AP*_25_ 82.62%. Although parameters decreased to 12.91M, performance dropped across all metrics.

#### 3.2.3 Class-wise instance segmentation performance and computational efficiency

To provide a more detailed evaluation across different rice organ categories, we report the class-wise *AP*, *AP*_50_, *AP*_25_, Precision_50_, and *AR*_50_ results of SPINet. We further analyze the model complexity and computational efficiency in terms of parameter count, FLOPs, inference throughput, latency, and GPU memory consumption. **Table 4** reports the organ-specific results obtained from the selected best SPINet checkpoint used in the main comparison.

**Table 4:** Class-wise instance segmentation performance.

Category	*AP*	*AP*_50_	*AP*_25_	Precision_50_	*AR*_50_
Overall	65.73	75.04	82.36	97.60	66.92
Grain	44.93	62.34	76.84	92.79	53.79
Leaf	63.47	71.77	77.50	100.00	63.64
Stem	88.78	91.02	92.74	100.00	83.33

The class-wise results show that SPINet achieved the highest performance for stem instances, with an *AP* of 88.78 and an *AR*_50_ of 83.33. In contrast, grain instances obtained an *AP* of 44.93 and an *AR*_50_ of 53.79, indicating that separating densely distributed, small-scale grain instances remains more challenging.

We further evaluated the computational complexity of SPINet. The model contains 34.36M parameters. For a fixed point-cloud input of 10,000 × 6, representing 10,000 points with XYZRGB features, the measured forward-pass profile was approximately 30.58 GFLOPs.

As summarized in **Table 5**, the archived validation logs recorded an end-to-end processing time of 0.1297 s per scene on one NVIDIA GeForce RTX 3090 with batch size 1, corresponding to a throughput of 7.71 scenes/s. The logged peak GPU memory was 20.65 GB.

**Table 5:** Computational complexity and logged efficiency of SPINet.

Method	Parameters	Input size	GFLOPs	Latency	Throughput	Peak memory
	(M)	(points × channels)		(s/scene)	(scenes/s)	(GB)
SPINet	34.36	10,000×6	30.58	0.1297	7.71	20.65

#### 3.2.4 Qualitative visualization

**Figure 7** presents qualitative comparisons of instance segmentation results. Conventional methods showed reduced segmentation quality in dense and occluded regions, where adjacent grains were frequently merged or boundaries became unclear. In contrast, SPINet produced more complete and better separated grain instances, particularly in regions with severe adhesion and inter-plant occlusion. These visual results are consistent with the quantitative improvements in *AP* and *AP*_50_, further indicating the advantage of the proposed global and sequence-neighborhood feature modeling and center prior-guided decoding strategy.

**Figure 7:**
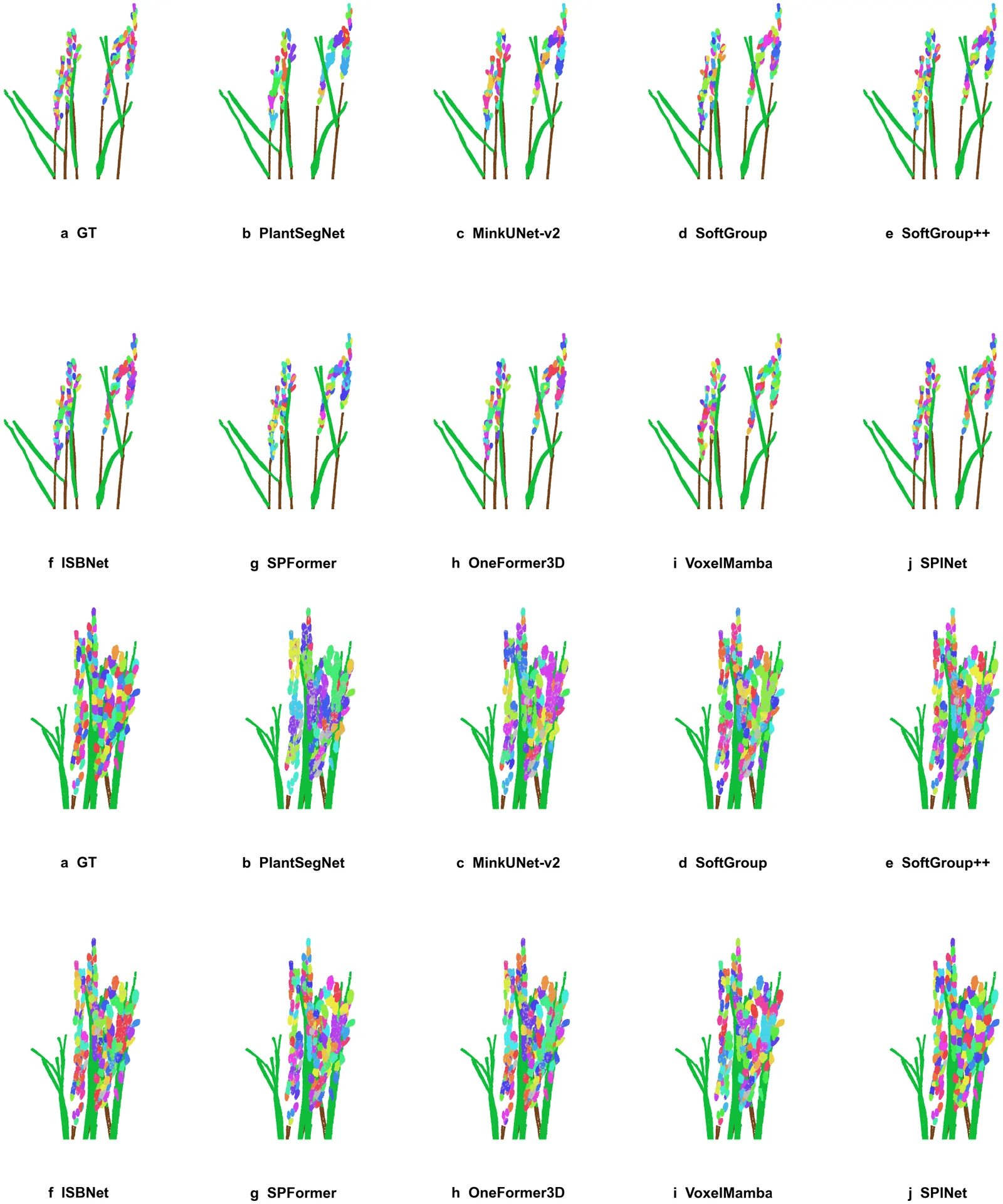
Qualitative comparison of 3D instance segmentation results on the GRD dataset. **(a)** Ground truth. **(b)** PlantSegNet. **(c)** MinkUNet-v2. **(d)** SoftGroup. **(e)** SoftGroup++. **(f)** ISBNet. **(g)** SPFormer. **(h)** OneFormer3D. **(i)** VoxelMamba. **(j)** SPINet. Different colors indicate different grain instances.

**Figure 8** further illustrate typical cases in which SPINet alleviates common segmentation errors. In dense grain clusters, the proposed method reduces erroneous instance merging and improves the separation of adjacent grains. In regions with unclear boundaries, SPINet preserves more distinct instance contours, which is critical for reliable grain counting and downstream phenotypic trait extraction.

**Figure 8:**
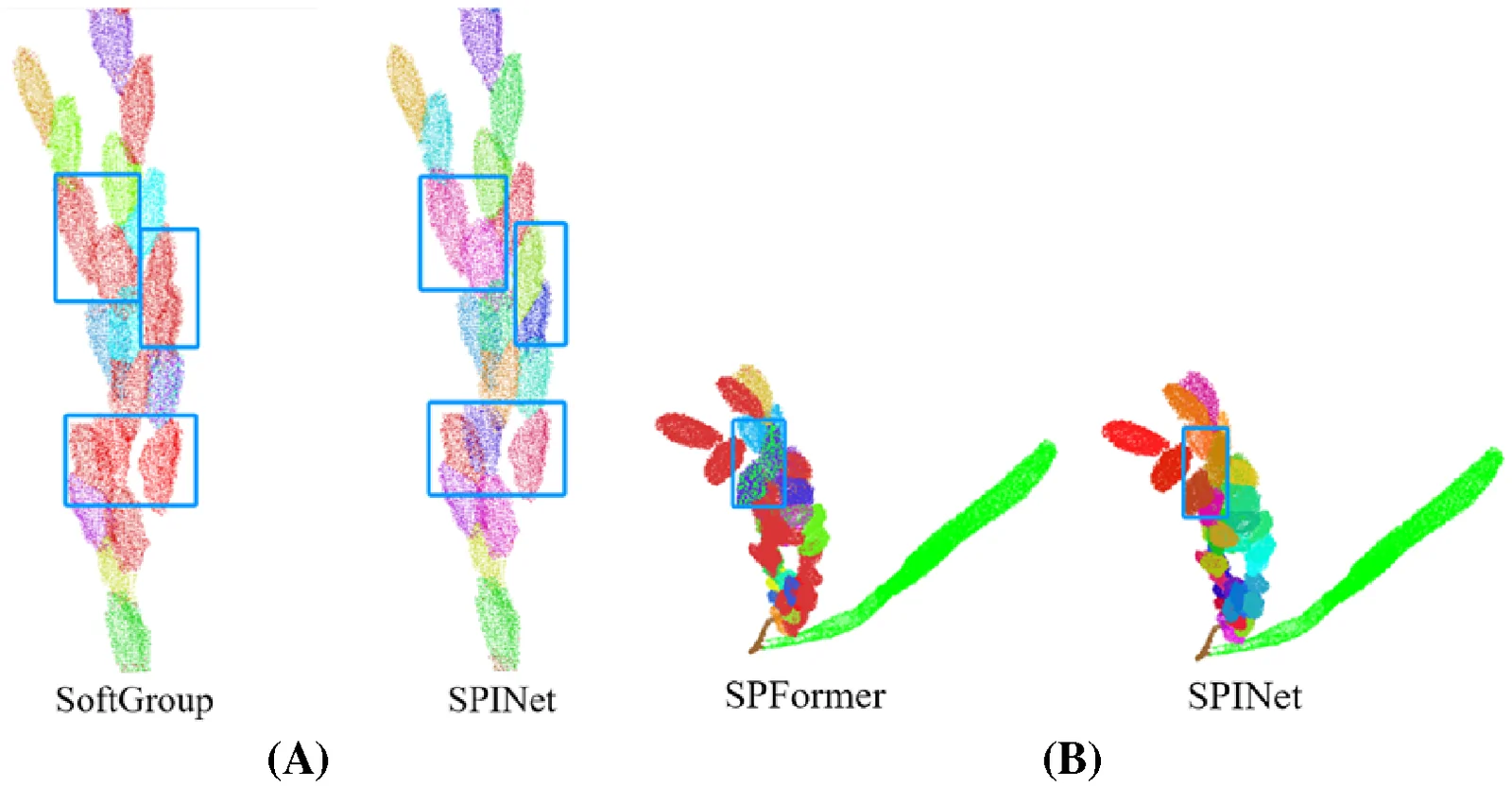
Typical error correction cases in dense rice grain instance segmentation. **(A)** Reduction of erroneous merging among adjacent grain instances. **(B)** Improved delineation of unclear grain boundaries.

### 3.3 Ablation study

To quantify the contribution of each component in SPINet, ablation experiments were conducted on the GRD validation set. The baseline model used Mamba2 as the backbone and achieved an *AP* of 57.69. As shown in **Table 6**, each proposed component contributed to the improvement of segmentation performance.

**Table 6:** Ablation results of the main components in SPINet.

RiceMamba2	Center decoder	DINO	*AP*	*AP*_50_	*AP*_25_
–	–	–	57.69	68.44	80.41
✓	–	–	62.00	74.11	83.04
–	✓	–	60.11	72.20	81.90
–	–	✓	60.66	71.94	**83.60**
✓	✓	✓	**65.73**	**75.04**	82.36

Bold values indicate the best results.

Effect of RiceMamba2. Introducing RiceMamba2 increased *AP* from 57.69 to 62.00, corresponding to an improvement of 4.31 percentage points. The improvement was particularly evident in *AP*_50_, which increased by 5.67 percentage points. This suggests that the integration of Mamba2-based long-range modeling and convolution-based feature enhancement improves the representation of both global plant structure and local grain boundaries.

Effect of the center-guided decoder. Adding the geometric center prior improved *AP* by 2.42 percentage points. This result indicates that explicit spatial anchors help the decoder localize potential instances and reduce the ambiguity of query matching in dense scenes. By guiding the model to refine masks around likely instance centers, the center-guided decoder can alleviate merging errors caused by inter-plant occlusion and organ adhesion.

Effect of DINO-based denoising. The DINO-based denoising strategy improved *AP* from 57.69 to 60.66. This gain suggests that auxiliary denoising queries provide more stable supervision during training, especially before Hungarian matching becomes reliable. By perturbing instance centers and category labels, the model learns to recover valid targets from noisy queries and suppress invalid ones, thereby improving robustness in dense small-instance segmentation.

When all components were combined, SPINet achieved the best overall performance, with an *AP* of 65.73 and an *AP*_50_ of 75.04. These results confirm the complementary contributions of RiceMamba2, the center-guided decoder, and DINO-based denoising to accurate 3D instance segmentation in multi-plant rice point clouds.

Effect of sequence-neighborhood enhancement. We further isolated the contribution of the convolutional branch over five independent runs. Removing this branch resulted in a validation *AP* of 58.07 ± 3.32, whereas enabling the shared convolutional branch increased *AP* to 61.29 ± 1.60, corresponding to an improvement of 3.22 percentage points. The logged validation latency increased from 0.1167 to 0.1263 s per scene, an increase of 0.0096 s per scene (8.23%). These results demonstrate the empirical benefit of the implemented sequence-neighborhood branch.

## 4 Discussion

### 4.1 Segmentation performance and phenotypic relevance

SPINet achieved an *AP* of 65.73% on the GRD dataset, demonstrating its effectiveness for 3D instance segmentation in dense multi-plant rice scenarios. Accurate grain instance segmentation provides the basis for non-destructive measurement of yield-related traits. Point cloud-based instance segmentation enables the direct extraction of individual grain instances in 3D space, allowing subsequent measurement of traits such as grain number, grain size, grain shape, and spatial distribution. These traits are valuable for germplasm evaluation, breeding material screening, and yield-related genetic studies such as genome-wide association studies (GWAS) (Visscher et al., 2017).

The performance advantage of SPINet mainly arises from its ability to address two key challenges in multi-plant rice scenarios: dense spatial arrangement and inter-plant occlusion. In such scenes, adjacent grains and panicles are often spatially close, visually similar, and partially adhered, making it difficult for conventional segmentation methods to distinguish instance boundaries. SPINet alleviates these issues by combining global spatial modeling with local geometric enhancement. The RiceMamba2 encoder captures long-range structural dependencies among plant organs, while the convolutional branch strengthens shortrange sequence-index correlations. In addition, the center prior-guided decoder provides explicit spatial anchors for instance queries, improving the separation of adjacent and adhered grains.

Although these results demonstrate the effectiveness of SPINet on GRD, they should be interpreted within the evaluated data domain. The stable acquisition environment reduces variations caused by illumination, plant motion, background clutter, and sensor conditions. Moreover, differences among rice cultivars in plant architecture, panicle morphology, grain density, and growth habit may alter both reconstruction quality and instance-segmentation difficulty. Consequently, the current experiments demonstrate within-dataset effectiveness, but do not establish generalization to field environments, genetically diverse rice populations, or different acquisition stages.

### 4.2 Implications for grain-level phenotypic extraction

Beyond segmentation accuracy, the practical value of SPINet lies in its ability to support downstream phenotypic trait extraction. Grain counting was performed directly from the predicted grain instances and compared with manually annotated ground-truth counts. As shown in **Figure 9**, the regression analysis yielded an *R*^2^ of 0.8532 and an RMSE of 1.78 grains per plant. The fitted regression equation was *y* = 0.94*x* + 1.70, indicating a strong association between the predicted and ground-truth counts. Error analysis are summarized in **Table 7**.

**Figure 9:**
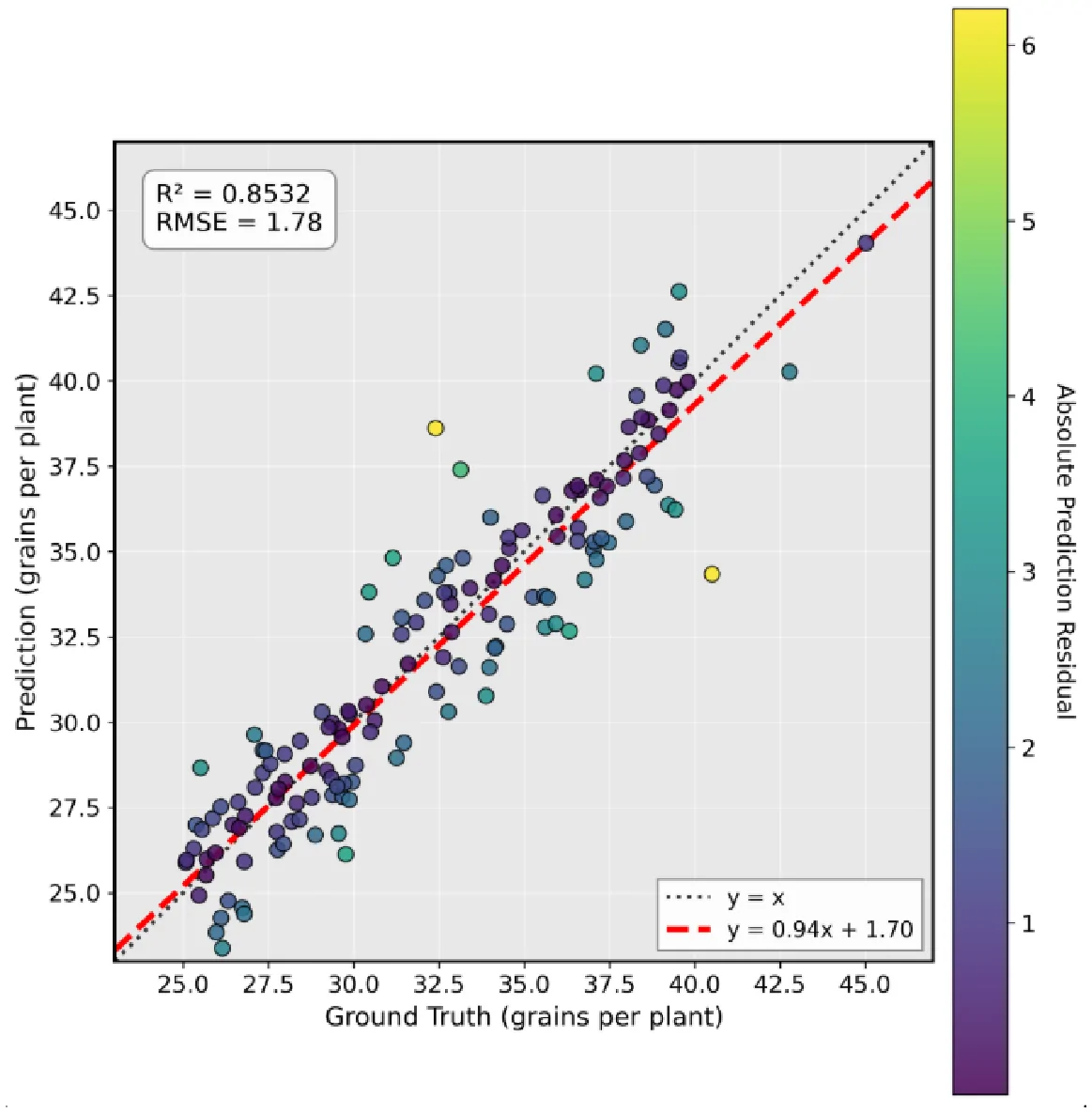
Regression analysis between predicted and ground-truth grain counts. The dotted line represents the identity relationship *y* = *x*, and the dashed line represents the fitted regression *y* = 0.94*x* + 1.70. The color of each point indicates the absolute counting residual.

**Table 7:** Grain-counting error analysis.

Metric	Value	Interpretation
*R* ^2^	0.8532	Regression agreement
RMSE	1.78	Grains per plant
MAE	1.45	Grains per plant
RAE	4.50%	Dataset-level error
MRE	4.53%	Mean relative error

The slope close to 1.0 suggests that SPINet maintains a stable proportional relationship across different grain densities. The positive intercept indicates a slight tendency toward overestimation, with an average bias of approximately 1–2 grains. From a breeding perspective, this level of accuracy is meaningful for preliminary screening of large-scale rice materials, where rapid and objective ranking of yield-related traits is often more important than exhaustive manual measurement of every individual grain.

Compared with conventional 2D image-based counting methods, 3D point cloud analysis provides an important advantage in handling occlusion and overlap. In 2D images, front-back occlusion and grain adhesion are difficult to resolve because depth information is not explicitly available. Although multi-view image fusion or post-processing strategies can partially alleviate this limitation, their performance remains constrained by incomplete spatial representation. By directly operating on reconstructed 3D point clouds, SPINet can exploit spatial depth and geometric context to better distinguish overlapping grains, which contributes to more reliable counting in dense multi-plant scenes.

The remaining counting errors mainly occurred in extremely dense grain clusters, where multiple grains were tightly adhered and their boundaries became ambiguous even in 3D space. In such cases, undersegmentation may occur, causing several neighboring grains to be predicted as a single instance. This observation indicates that the main bottleneck of the current method lies in resolving fine-grained boundaries under severe adhesion and occlusion. Future improvements may therefore benefit from higher-resolution reconstruction, stronger boundary-aware supervision, or topology-aware refinement modules.

### 4.3 Cross-dataset evaluation on RicePanicle

To assess cross-dataset generalization, we directly evaluated OneFormer3D and SPINet on 20 external rice-panicle point clouds, hereafter referred to as RicePanicle. These samples were obtained from the public dataset and code release accompanying the Panicle-3D project Gong et al. (2021).

Both models retained the weights learned from GRD. No RicePanicle samples or annotations were used for retraining, fine-tuning, model selection, or *post-hoc* label alignment. Because the semantic labels provided by RicePanicle do not define grain-level instances corresponding to the GRD instance label space, we performed inference directly on the point clouds without enforcing a strict correspondence between the two label systems.

As shown in **Figure 10**, the predictions reveal a clear qualitative difference between the two models. In the displayed RicePanicle samples, OneFormer3D tended to merge spatially adjacent grains into larger regions, particularly in densely clustered portions of the panicle. In comparison, SPINet visually preserved finer separation between neighboring grains and produced more coherent grain-level regions. These observations indicate that SPINet retained a stronger ability to distinguish individual grains under the geometric and acquisition-domain differences introduced by RicePanicle.

**Figure 10:**
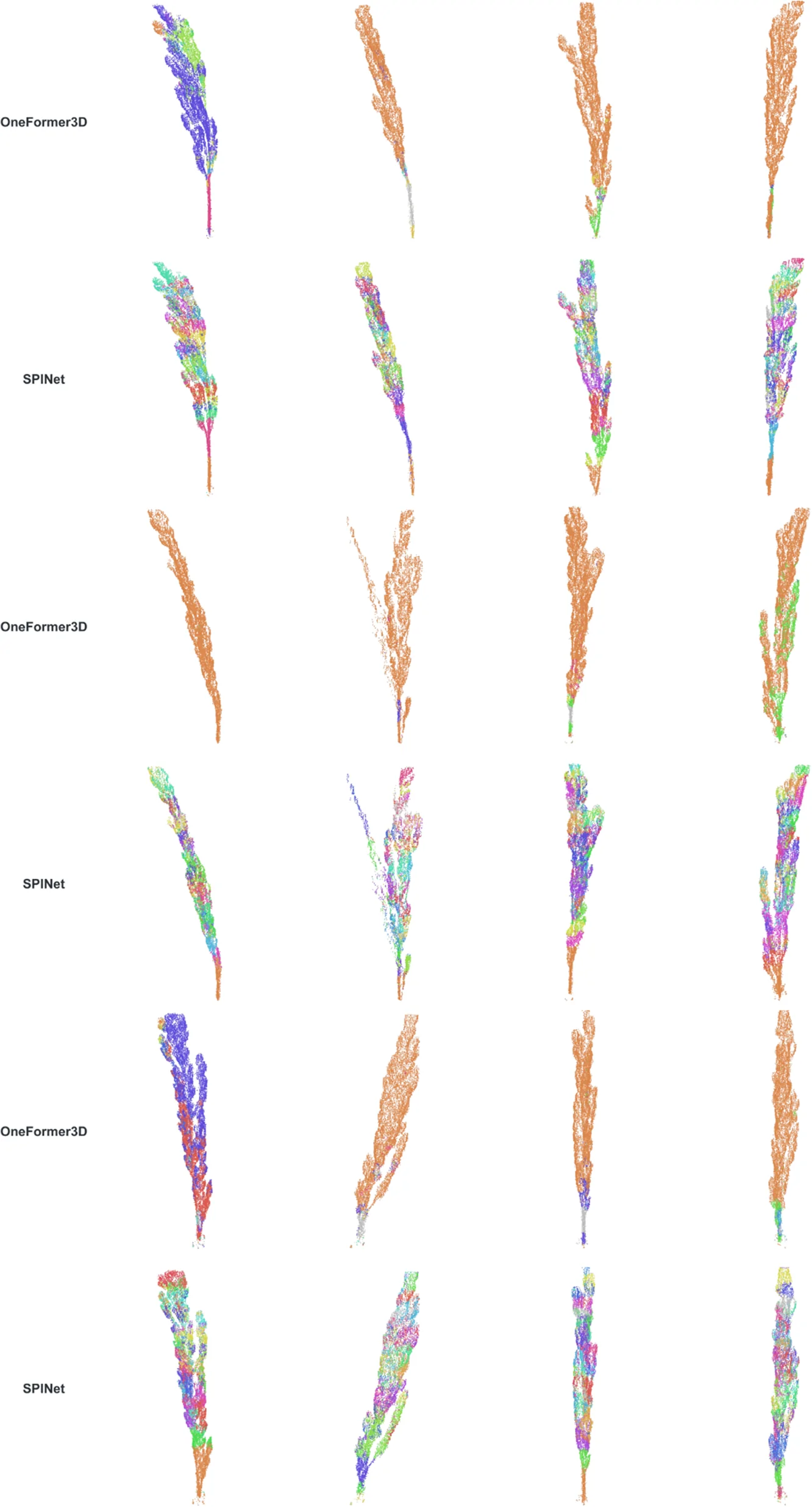
Qualitative cross-dataset comparison between OneFormer3D and SPINet on RicePanicle. Colors distinguish predicted instances within each result.

To further characterize the qualitative results, we quantified the semantic composition of the retained instance predictions across the 20 RicePanicle scenes (**Table 8**). With a common minimum of 30 exclusively assigned points per predicted instance, OneFormer3D produced 105 retained regions, of which 95 (90.5%) were classified as leaf, 6 (5.7%) as stem, and only 4 (3.8%) as grain. In contrast, SPINet produced 895 retained regions, including 885 (98.9%) classified as grain, 9 (1.0%) as leaf, and 1 (0.1%) as stem. The strong leaf bias of OneFormer3D is consistent with the qualitative observation that many panicle structures were not resolved as individual grains, whereas SPINet maintained a grain-dominant response under the same cross-dataset inference setting.

**Table 8:** Semantic composition of retained instance predictions on the 20 RicePanicle scenes.

Method	Grain	Leaf	Stem	Total
OneFormer3D	4 (3.8%)	95 (90.5%)	6 (5.7%)	105
SPINet	885 (98.9%)	9 (1.0%)	1 (0.1%)	895

Values in parentheses are percentages of all retained instance regions predicted by the corresponding method. A common minimum of 30 exclusively assigned points was used.

### 4.4 Potential influence of occlusion and reconstruction quality

Because SPINet operates on point clouds reconstructed from multi-view images, its segmentation performance may also depend on the quality of the upstream 3D reconstruction. To illustrate several possible failure conditions, **Figure 11** presents three representative multi-plant cases constructed from GRD point clouds. Limited azimuth coverage can leave rear-facing grain surfaces weakly constrained, while sparse view sampling reduces the number of effective ray intersections. When severe inter-plant occlusion is combined with low parallax, the depth and geometry of overlapping grains become more difficult to reconstruct reliably.

**Figure 11:**
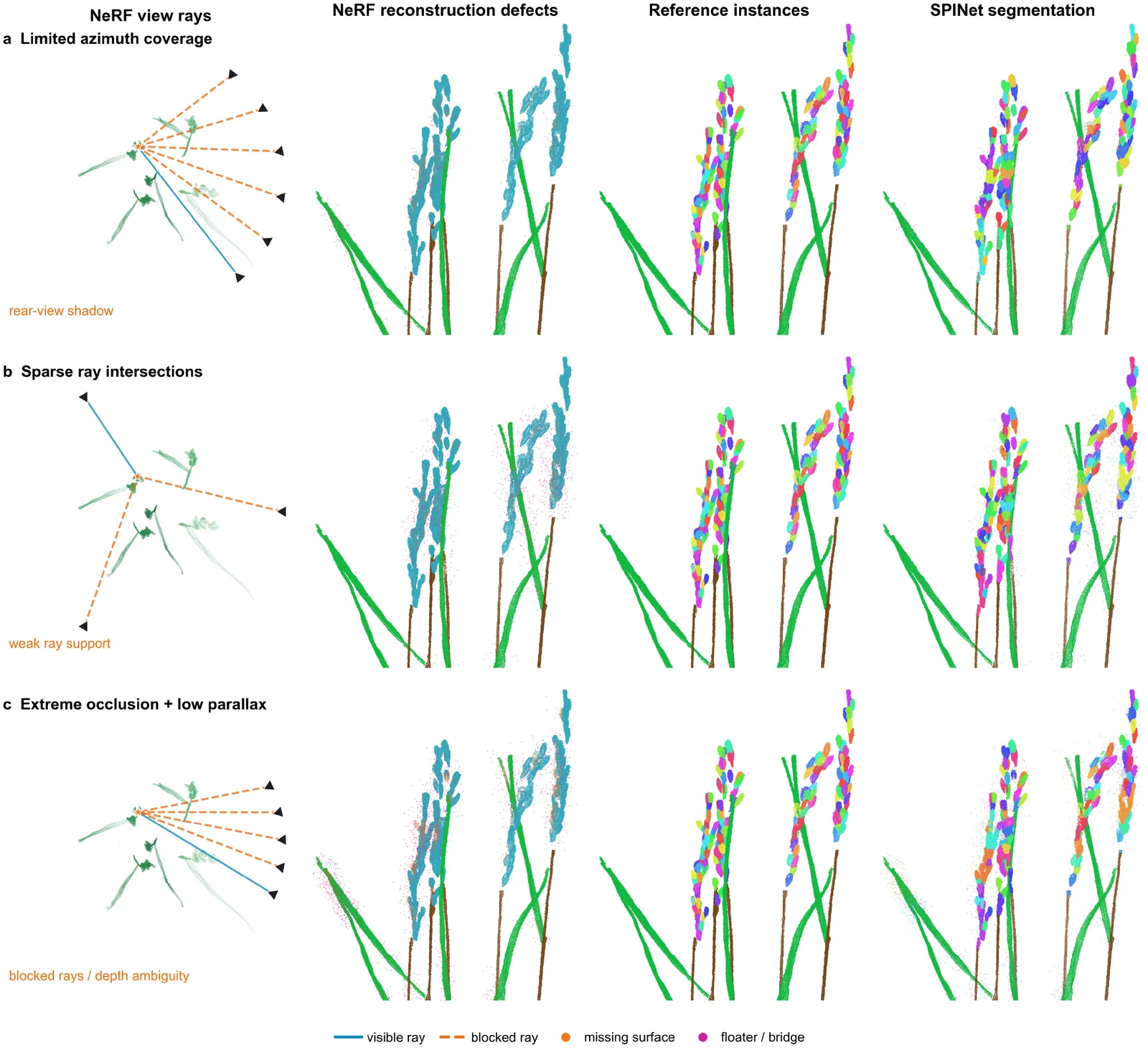
Illustration of the potential influence of multi-view coverage and inter-plant occlusion on NeRF reconstruction and subsequent SPINet segmentation. The three rows show **(A)** limited azimuth coverage, **(B)** sparse ray intersections, and **(C)** extreme occlusion combined with low parallax. Blue lines represent visible viewing rays, whereas orange dashed lines represent rays blocked by foreground plant structures. Missing surfaces and floating or bridging artifacts indicate possible reconstruction defects. The reference instances and corresponding SPINet segmentation results illustrate how incomplete geometry can affect grain-level instance separation. These cases provide a qualitative mechanism illustration and are not used for quantitative evaluation.

Future work will therefore evaluate viewpoint coverage and reconstruction quality in a controlled manner to clarify their effects on downstream segmentation and phenotypic measurements.

### 4.5 Limitations and future work

Although SPINet showed promising performance in controlled multi-plant rice scenarios, several limitations remain. First, the GRD dataset was collected under stable indoor conditions, whereas practical breeding and field phenotyping environments are affected by variable illumination, wind-induced plant movement, complex backgrounds, weeds, and sensor noise. These factors may influence 3D reconstruction quality and segmentation stability, indicating that further validation under field conditions is necessary.

Second, the current dataset covers a limited range of rice materials and growth conditions. Rice varieties with different plant architectures, panicle types, and grain densities may present different segmentation challenges. Therefore, expanding the dataset to include more diverse germplasm resources, growth stages, and planting densities will be important for improving the generalizability of SPINet in breeding applications.

Future work will focus on collecting field-scale multi-season rice point cloud datasets, improving domain adaptation across varieties and environments, and developing boundary-aware modules for densely adhered grains. These efforts may further enhance the robustness of SPINet and support its application in high-throughput rice phenotyping and yield-related trait evaluation.

## 5 Conclusion

This study proposed SPINet, an end-to-end point cloud instance segmentation framework for 3D phenotypic analysis in multi-plant rice scenarios. The method was designed to address two major challenges in dense rice point clouds: the difficulty of jointly capturing global spatial context and local geometric details, and the ambiguous instance attribution caused by inter-plant occlusion and organ adhesion. To this end, SPINet integrates three key components. First, the RiceMamba2 encoder combines the long-range dependency modeling capability of Mamba2 with a parallel convolutional branch for local structure enhancement, enabling effective representation of both global plant organization and short-range sequence-neighborhood cues. Second, the center prior-guided instance decoder introduces geometric center information as explicit spatial anchors, improving the separation of adjacent and adhered instances in dense scenes. Third, a DINO-inspired denoising training strategy is incorporated to stabilize query learning and improve convergence.

Experiments on the self-constructed Grouped Rice Dataset (GRD) demonstrated the effectiveness of the proposed method. SPINet achieved an Average Precision (*AP*) of 65.73% for 3D instance segmentation. The grain-counting analysis yielded an *R*^2^ of 0.8532 and an RMSE of 1.78 grains per plant, indicating its potential for downstream yield-related phenotypic trait extraction. However, the present evidence is restricted to a limited range of rice materials acquired at a single stage in a stable phenotyping environment. Generalization to diverse cultivars, field conditions, different sensors, growth stages, and reconstruction pipelines has not yet been established. Overall, this study provides a robust and effective framework for automated 3D rice phenotyping in complex multi-plant environments.

## Data Availability

The original contributions presented in the study are included in the article/supplementary material. Further inquiries can be directed to the corresponding author.
