# Correction: SPINet: structure-enhanced point cloud instance segmentation network for multi-plant rice phenotyping

**DOI:** 10.3389/fpls.2026.1998555

**Published:** 2026-09-21

**Authors:** Wentao Song, Fang Qu, Yuwei Hao, He Huang, Caiguo Tang, Youqiang Sun, Junqing Zhang

**Affiliations:** 1 Hefei Institutes of Physical Science, Chinese Academy of Sciences, Hefei, China; 2 University of Science and Technology of China, Hefei, China; 3 Institute of Hefei Artificial Intelligence Breeding Accelerator Co. Ltd., Hefei, China

**Keywords:** 3D instance segmentation, grain counting, Mamba2, multi-plant scene, point cloud, rice phenotyping, spatial feature learning

There was a mistake in **Figure 10** as published. The image displayed for **Figure 10** was inadvertently duplicated from Figure 5. The corrected **Figure 10** appears below.

**Figure 10:**
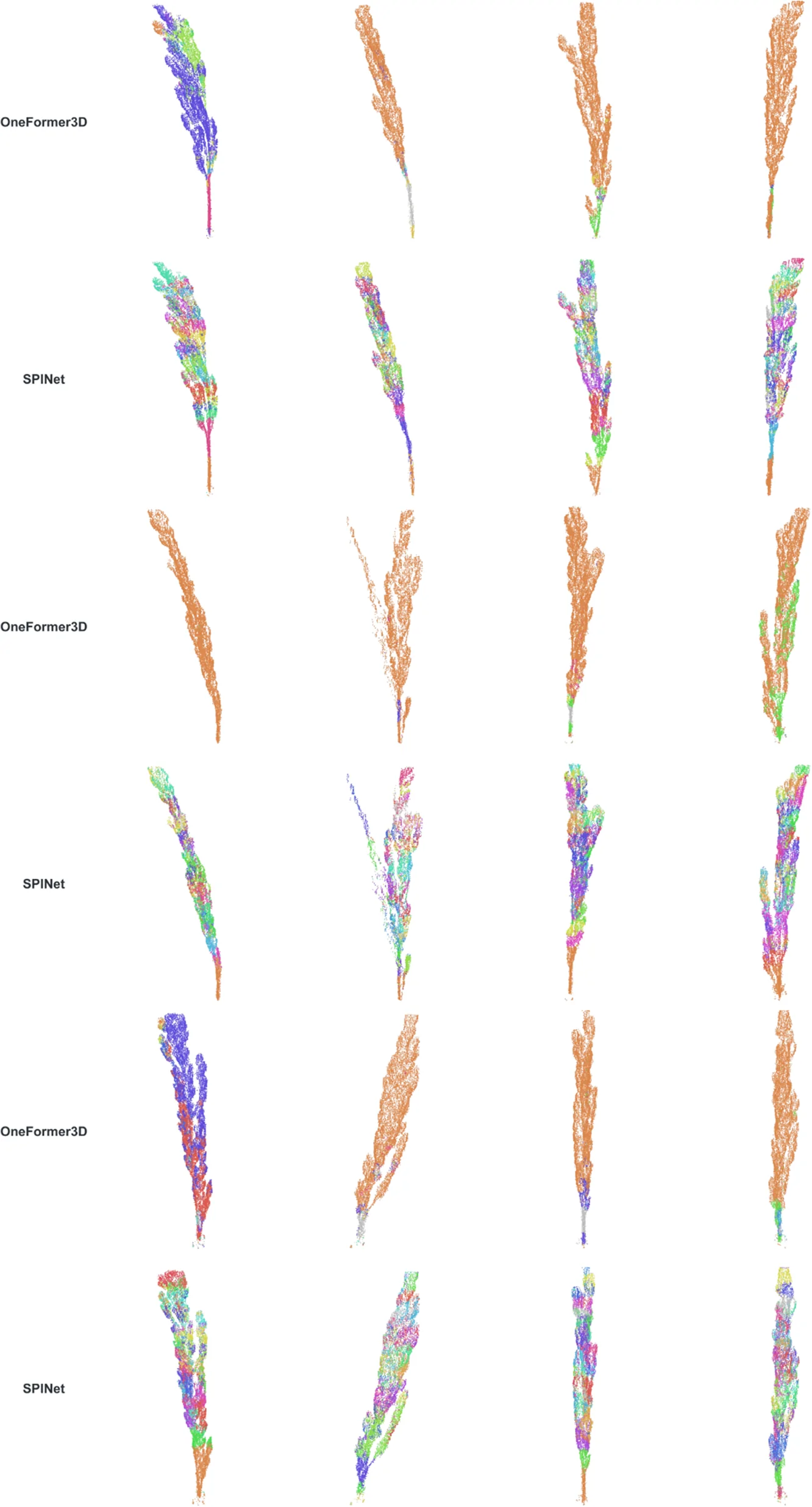
Qualitative cross-dataset comparison between OneFormer3D and SPINet on RicePanicle. Colors distinguish predicted instances within each result.

The original version of this article has been updated.

